# Colon-Targeted astragalus polysaccharide nanoparticles prevent NAFLD-Driven hepatocarcinogenesis via microbiota remodeling and NF-κB Inhibition

**DOI:** 10.1186/s13046-025-03608-z

**Published:** 2025-12-20

**Authors:** Dan Liu, Runtian Li, Mingzhu Li, Ying Liang, Zhao Wang, Yang Sun, Pengling Ge

**Affiliations:** 1 Department of Biology, College of Basic Medicine, Heilongjiang University of Chinese Medicine, Harbin, 150040 China; 2 Shanghai Jinghe Biopharmaceutical Co., Ltd, Shanghai, 201406 China; 3 Department of Pharmacology, College of Basic Medicine, Heilongjiang University of Chinese Medicine, Harbin, 150040 China

**Keywords:** Hepatocellular carcinoma, Astragalus polysaccharide, Gut–liver axis, NF-κB signaling, Short-chain fatty acids, Colon-targeted nanoparticles

## Abstract

Hepatocellular carcinoma (HCC), often arising from liver fibrosis in nonalcoholic fatty liver disease (NAFLD), remains a leading cause of cancer-related death. Targeting the gut–liver axis offers new therapeutic opportunities to prevent this progression. In this study, colon-targeted chitosan/pectin-based nanoparticles loaded with Astragalus polysaccharide (APs-CS/PT-NPs) were developed to modulate gut microbiota and inhibit liver tumorigenesis. The nanoparticles exhibited robust physicochemical stability and pH-responsive release. In vivo, oral administration of APs-CS/PT-NPs attenuated hepatic steatosis, reduced inflammatory cytokines, and suppressed NAFLD-induced HCC development. 16 S rRNA sequencing revealed restoration of microbial diversity and enhanced production of short-chain fatty acids, especially acetate. Mechanistically, transcriptomic profiling and functional analysis identified acetate as a key mediator, acting via G-protein-coupled receptor 43 (GPR43) to inhibit the NF-κB pathway. These results highlight the therapeutic potential of APs-CS/PT-NPs in modulating the gut–liver axis, rebalancing intestinal microbiota, and suppressing pro-inflammatory signaling. This nanoparticle-based strategy offers a promising food-derived preventive intervention for liver fibrosis–HCC transition.

**Graphical Abstract:**

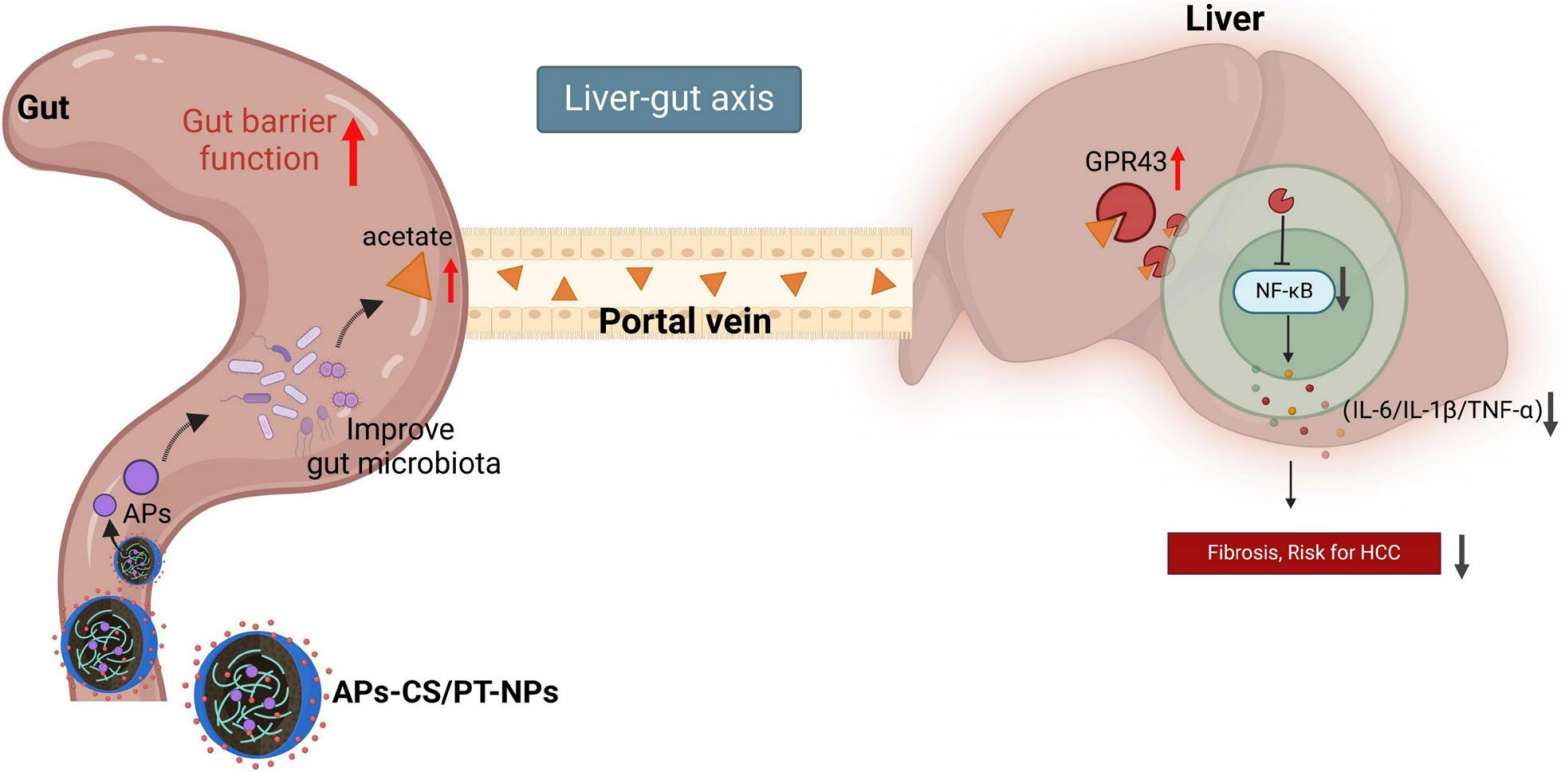

**Supplementary Information:**

The online version contains supplementary material available at 10.1186/s13046-025-03608-z.

## Introduction

Nonalcoholic fatty liver disease (NAFLD) represents one of the most common chronic liver disorders globally, affecting over one-quarter of the population [1]. The pathological progression of NAFLD involves several distinct stages, including non-alcoholic fatty liver (NAFL), non-alcoholic steatohepatitis (NASH), liver fibrosis, and ultimately hepatocellular carcinoma (HCC) [2]. HCC is a highly malignant liver cancer with increasing incidence and poor survival rates [3]. The development of NAFLD-associated HCC (NAFLD-HCC) involves a multifactorial pathogenesis driven by lipid metabolism imbalance, persistent inflammation, oxidative stress, and alterations in the immune microenvironment [4–7]. Conventional HCC therapies, including surgical resection, chemotherapy, targeted therapy, and immunotherapy, provide limited efficacy because most cases are diagnosed at advanced stages and exhibit resistance to existing treatments. Early intervention and exploration of novel therapeutic strategies are essential [8–10].

Recent findings highlight the gut microbiota (GM) as a key regulator in NAFLD progression, primarily through its modulation of liver homeostasis along the gut–liver axis [11, 12]. Microbial dysbiosis disrupts short-chain fatty acid (SCFA) metabolism, alters bile acid signaling, and activates pro-inflammatory pathways, collectively promoting lipid accumulation, aggravating hepatic inflammation, and facilitating HCC development [13–17]. Consequently, targeting the gut-liver axis, especially by improving the composition of the GM and its metabolites, has emerged as one of the novel strategies for preventing and treating NAFLD-HCC.

The gut-liver axis refers to the bidirectional communication system between the gut and liver, mediated through portal vein circulation, bile acid metabolism, and immune modulation. The axis is essential for maintaining hepatic homeostasis and controlling disease progression. Disruption of gut microbial balance impairs intestinal barrier integrity, enabling bacterial metabolites and endotoxins such as lipopolysaccharides (LPS) to enter the portal circulation and trigger hepatic inflammation [18–20]. Furthermore, metabolic products from the GM, including SCFAs, bile acids, and tryptophan derivatives, regulate liver metabolism and immune responses through activation of nuclear receptors such as farnesoid X receptor (FXR) and peroxisome proliferator-activated receptors (PPARs), as well as G-protein-coupled receptors (GPRs) [21–23].

In recent years, SCFAs, particularly acetate, have demonstrated significant roles in modulating the gut-liver axis. Acetate regulates the Nuclear Factor kappa B (NF-κB) signaling pathway by binding to the G-protein-coupled receptor 43 (GPR43), thereby inhibiting inflammation and improving lipid metabolism [24, 25]. However, effectively increasing the endogenous levels of acetate and ensuring its stable therapeutic effect on HCC remains a major challenge in current research. Given this background, exploring novel delivery strategies that can target the GM and enhance acetate levels may offer new intervention approaches for the prevention and treatment of NAFLD-HCC.

Currently, most drug therapies for NAFLD-HCC face challenges such as low bioavailability, poor targeting, and significant side effects, which limit their clinical application [10, 26]. In recent years, nanoparticle-based delivery systems have emerged as a promising approach in drug delivery due to their advantages, including high drug loading capacity, targeted delivery, sustained release, and improved bioavailability [27–30]. Chitosan (CS) is a chitin-derived polysaccharide that is biodegradable, biocompatible, non-toxic, and exhibits strong mucoadhesive properties. Its insolubility under normal colonic pH conditions prevents premature drug release and enables targeted delivery to inflamed sites, making it a widely used natural nanocarrier. However, the acidic gastric environment can protonate the free amino groups in CS, disrupting its structure and limiting its ability to transport drugs through the stomach [31]. In contrast, pectin (PT) is a biocompatible and mucoadhesive polysaccharide that can be degraded by GM [32]. The physicochemical properties of PT remain stable under gastrointestinal conditions, allowing it to pass through the stomach and reach diseased regions of the colon [33]. Nevertheless, the high water solubility of PT alone fails to prevent premature drug release before reaching the colon. To overcome these limitations, a CS/PT composite nanocarrier was designed with the expectation that its pH responsiveness and microbiota-mediated degradability would enable site-specific drug release within the colon.

Astragalus polysaccharides (APs), the principal macromolecular bioactive component of *Astragalus membranaceus* (AR), have shown significant therapeutic potential in treating various diseases, including neurological disorders, cardiovascular diseases, diabetes, and cancer. Previous studies have indicated that APs can alleviate diseases induced by a high-fat diet (HFD) through GM modulation [34–37]. However, due to its large molecular size, bulky structure, and negative charge, APs exhibit low bioavailability, limiting their clinical application.

To address these challenges, the present study designed an oral colon-targeted delivery system (OCDDS) to achieve precise delivery of APs to the colon and enhance their therapeutic efficacy. Specifically, we developed an oral colon-targeted delivery system based on APs-CS/PT-NPs to modulate the gut-liver axis and improve HCC progression. This nanoplatform exhibits colon-targeting properties, enabling controlled and sustained drug release under intestinal pH conditions, while effectively modulating the GM. Through this system, we aimed to enhance the ability of APs to regulate GM, increase acetate/acetyl ester levels, influence the gut–liver axis, and ultimately ameliorate the development and progression of HCC. We constructed a NAFLD-HCC mouse model to systematically assess the impact of APs-CS/PT-NPs on GM structure, acetate production, GPR43 signaling, and NF-κB-mediated inflammatory responses. Additionally, 16 S rRNA sequencing, targeted metabolomics, and transcriptomics were employed to elucidate the underlying mechanisms. The anti-HCC effects were further validated using in vitro cell models and in vivo xenograft models. The innovation of this study lies in the use of nanotechnology to achieve targeted modulation of the gut-liver axis. By improving microbiota structure, enhancing acetate levels, and regulating GPR43 receptors and NF-κB signaling, we aim to inhibit HCC progression. The findings provide a novel precision intervention strategy for the prevention and treatment of NAFLD-HCC and offer new perspectives on modernizing traditional Chinese medicine through nanoparticle-based delivery systems. The proposed approach holds significant potential for early prevention and precision therapy of HCC and represents an important step toward the advancement of personalized medicine.

## Materials and methods

### Preparation of APs-CS/PT-NPs

The preparation of APs-CS/PT-NPs was adapted with modifications from Kang Yuzhao et al. [38]. First, 20 mg of APs (APs, A860847, Macklin) was dissolved in 20 mL of deionized water to obtain a 1 mg/mL solution as the negatively charged component. CS (C299272, Aladdin; 40 mg) was dissolved in 10 mL of 1% citric acid (pH 4.0–5.0) to yield a 4 mg/mL positively charged solution. PT (P7536, Sigma; 20 mg) was dissolved in 20 mL PBS (pH 6.8) to form a 1 mg/mL stabilizing solution. Under magnetic stirring, the CS solution (4 mg/mL) was added dropwise to the APs solution (1 mg/mL) at a stirring speed of 300–500 rpm for 5–10 min to form preliminary nanoparticles (NPs) through electrostatic interactions. Then, the PT solution (1 mg/mL) was slowly added, further enhancing the stability of the particles through electrostatic interactions and hydrogen bonding, and stirring continued for an additional 10 min. Subsequently, a 1 mg/mL tripolyphosphate (TPP, 238503, Sigma) solution was introduced to crosslink CS and stabilize the NPs. The mixture was stirred for 30 min, maintaining the component ratio CS: APs: PT: TPP = 4:4:2:1. The NPs were separated by ultracentrifugation at 4000 rpm for 20 min using a desktop low-speed centrifuge (TD5A-WS, China), washed with deionized water, and freeze-dried. The solid complex weight was determined by weighing the resulting precipitate.

## Measurement of encapsulation efficiency (EE)

The quantification of unbound APs in the supernatant and weakly adsorbed APs on the nanoparticle surface was performed by sequential centrifugation and two methanol washes, following and slightly modifying the method reported by Ran Xin et al. [39]. High-performance liquid chromatography (HPLC) was used for detection with the following conditions: Waters Symmetry C18 column (250 mm × 4.6 mm, 5 μm); mobile phase: 0.02% formic acid aqueous solution–acetonitrile, gradient elution (0–30 min, 16%–49% acetonitrile; 30–50 min, 49%–16% acetonitrile); column temperature: 30 °C; injection volume: 40 µL; flow rate: 1.0 mL/min; evaporative light scattering detector: gas pressure 275.79 kPa (40.0 psi), drift tube temperature 65 °C. All analytes achieved baseline separation, and the theoretical plate number was >4000. The amount of APs in the samples was determined by comparing the peak area with that obtained from a similarly treated standard curve. The EE was calculated as follows:

 

$$\:\%\text{E}\text{E}\text{=}\frac{\text{Total APs added-}\text{U}\text{nbound APs}}{\text{Total APs added}}\times100\%$$

### Characterization of APs-CS/PT-NPs

The preparation and optimization of APs-CS/PT-NPs were adapted with appropriate modifications from the method reported by Kang Yuzhao et al. [38]. The particle size, polydispersity index (PDI), and zeta potential of APs-CS/PT-NPs were measured using Dynamic Light Scattering (DLS) technology (Nano ZS, Malvern, UK). After dilution, APs-CS/PT-NPs were deposited onto copper grids coated with a 200-mesh carbon film, followed by drying. The morphology of the APs-CS/PT-NPs was then analyzed using a Transmission Electron Microscope (TEM) (HTACHI, H-7650, Japan).

### In vitro release analysis

The in vitro release of APs from NPs was investigated using the dialysis bag diffusion method (12,000 Da cellulose acetate dialysis bag, Sigma, USA) [33]. One milliliter of APs-CS/PT-NPs was placed into the dialysis bag and suspended in 20 mL of HCl solution at pH 2.0 for 2 h to simulate the gastric environment. Subsequently, the dialysis bag was suspended in 20 mL of PBS at pH 6.8 for 4 h to mimic the small intestine environment. The dialysis bag was then incubated in 20 mL of PBS (pH 7.4, containing 20% mouse cecal filtrate) at 37 °C with stirring for 8 h to simulate the colon environment. At the end of each simulation phase, samples were collected, and the supernatants were separated by centrifugation for APs analysis. Release tests were performed in triplicate at each time point. To evaluate pH sensitivity and microbial responsiveness, nanoparticle morphology was examined by transmission electron microscopy (TEM), and particle size distribution was analyzed under different simulated conditions. Drug release in the gastric phase, governed by diffusion and matrix erosion, followed first-order kinetics, expressed as: ln(1 − *Qt*​/*Q*∞​) = − *kt*, where (Qt​/Q∞) represents the cumulative release fraction at time t, and k is the release rate constant.

### Long-term stability testing

Long-term stability testing was performed with modifications based on Kang Yuzhao et al. [38]. APs-CS/PT-NPs were stored at 4 °C, and their average particle size and zeta potential were measured every 7 days. After 35 days, nanoparticle morphology was analyzed by TEM.

### Fourier transform infrared spectroscopy (FTIR)

FTIR analysis was conducted with modifications according to the method of Zhang Peng et al. [40]. Briefly, KBr powder was ground and dried at 105 °C for 4 h, then stored in a desiccator. Dried nanoparticle powder (1–2 mg) was mixed with 100-fold mass of KBr, ground to ~ 2 μm, and compressed into thin pellets at 15 MPa. FTIR spectra were recorded with a resolution of 4 cm⁻¹, 32 scans per sample, over the wavenumber range of 400–4000 cm⁻¹.

### Cell culture

Human HCC cell line Huh-7 (SNL-085) was obtained from ATCC and cultured in DMEM (30–2002, ATCC) supplemented with 10% fetal bovine serum (FBS, 30–2020, ATCC) and 1% penicillin–streptomycin (P/S, PB180120, Pronoase). Normal hepatocyte line MIHA (CL0469) was purchased from Fenghui Biotechnology and maintained in RPMI-1640 medium (PM150110, Pronoase) with 10% FBS and 1% P/S. All cells were cultured at 37 °C in a humidified atmosphere containing 5% CO₂ and 95% air.

### Cell treatment and grouping

Lentiviral particles carrying sh-NC or sh-GPR43 were packaged in HEK-293T cells (CRL-11268, ATCC) using the pLenti6/BLOCK-iT™-DEST vector (K494300, ThermoFisher). Viral supernatants were collected 48 h post-transfection, concentrated by Genechem (Shanghai, China), and used to transduce Huh-7 cells at a multiplicity of infection (MOI) of 5. After 48 h, cells were selected with puromycin (10 µg/mL; HY-K1057, MCE) for 1 week to establish stable knockdown lines [41, 42]. Transfection efficiency was confirmed by Western blot (WB) 48 h after infection. Huh-7 and MIHA cells were treated with 50 mM sodium acetate for 72 h [43], with distilled water as control. Experimental groups included: Vehicle (control), Acetate, Vehicle + sh-NC (Huh-7 infected with sh-NC), Acetate + sh-NC (Huh-7 infected with sh-NC and treated with acetate), Vehicle + sh-GPR43 (Huh-7 infected with sh-GPR43), and Acetate + sh-GPR43 (Huh-7 infected with sh-GPR43 and treated with acetate).

### 3-(4,5-Dimethylthiazol-2-yl)−2,5-diphenyltetrazolium bromide (MTT) assay

Forty-eight hours after lentiviral infection, cells were cultured at 37 °C for 0, 24, 48, and 72 h. The medium was then replaced with complete medium containing 0.5 mg/mL MTT (C0009S, Beyotime, China) and incubated for 4 h. Afterward, 150 µL dimethyl sulfoxide (DMSO; ST1276, Beyotime, China) was added, and plates were shaken in the dark for 10 min. Absorbance was recorded at 570 nm using a microplate reader (800TS, BioTek, Agilent, USA). Preliminary cytotoxicity screening confirmed that 50 mM sodium acetate provided optimal efficacy and safety; this concentration was used for subsequent in vitro assays.

### Colony formation assay

Cells were seeded in 35 mm dishes (400 cells/dish) and cultured at 37 °C for approximately two weeks. Colonies were washed twice with PBS, fixed with 4% paraformaldehyde (PFA) for 20 min, and stained with Wright–Giemsa solution (C0131, Beyotime, China) for 5 min. Colonies containing > 50 cells were counted.

### Flow cytometry

Apoptosis was evaluated following the method of Chunfei Wu et al. [33]. Cells were stained using an Annexin V–FITC/propidium iodide (PI) apoptosis detection kit (Beyotime, Shanghai, China). Briefly, cells were incubated in 100 µL binding buffer containing 1 µL Annexin V–FITC and 10 µg/mL PI for 15 min in the dark, followed by the addition of 400 µL binding buffer and analysis by flow cytometry.

For cell cycle analysis, cells were washed twice with PBS and fixed in pre-chilled 70% ethanol at 4 °C for 4 h. After two additional PBS washes, cells were incubated in PBS containing 0.2% Triton X-100 and 10 µg/mL RNase at 37 °C for 30 min, then resuspended in 400 µL PI solution (50 µg/mL; HY-D0815, MCE, China) and incubated in the dark (4 °C, 30 min). Cell cycle distribution was analyzed using a BD FACSVerse flow cytometer (BD Biosciences, USA) and its accompanying software.

### Wound healing assay

Cell migration was assessed by a scratch assay. Cells were seeded into 6-well plates at 1 × 10⁵ cells/mL and grown in complete medium until ~ 75% confluence. A sterile 100 µL pipette tip was used to create a straight scratch perpendicular to the cell layer, ensuring comparable scratch widths among groups. After scratching, the medium was removed, cells were washed twice with PBS, and cultured in serum-free medium. Images were taken at 0 h and after incubation at 37 °C to evaluate wound closure.

### Transwell migration assay

Cell migration was assessed using 24-well Boyden chambers with 8.0 μm pore polycarbonate membranes (Corning, USA). Cells were trypsinized and seeded in the upper chamber at 1 × 10⁵ cells/well in 100 µL serum-free medium, while the lower chamber contained 600 µL complete medium as a chemoattractant. After 24 h of incubation at 37 °C, non-migrated cells on the upper membrane surface were removed with cotton swabs. Cells that had migrated to the underside were fixed with 4% PFA, stained with crystal violet, and photographed using an inverted fluorescence microscope. Migrated cells were counted in five randomly selected microscopic fields per membrane.

### Animal sources and ethical statement

Two-week-old male C57BL/6J wild-type mice (219) and four-week-old BALB/c Nude mice (401) were purchased from Beijing Vital River Laboratory Animal Technology Co., Ltd. Six-week-old male germ-free C57BL/6 mice were obtained from Gnotobio. All mice were housed in pathogen-free, environmentally controlled barrier facilities with a 12-hour light/dark cycle, at a temperature of 24 ± 1 °C and humidity of 50 ± 10%. Food and water were provided ad libitum.

All animal procedures complied with the guidelines of the Institutional Animal Ethics Committee, College of Basic Medicine, Heilongjiang University of Chinese Medicine (Approval No. 2025050915). Animal handling and care followed internationally recognized welfare standards, with efforts made to minimize suffering and reduce animal use. Proper care and humane disposal were ensured throughout and after all experiments.

### In vivo safety evaluation

The biocompatibility of the nanocarrier, a key parameter for clinical translation, was assessed following the modified method of Long Huang et al. [44]. Normal mice were orally administered the respective formulations, with an untreated group (NC) as control. Three months after administration, all mice were euthanized by cervical dislocation. Blood samples were collected and centrifuged at 5000 rpm for 10 min to measure serum levels of aspartate aminotransferase (AST), alanine aminotransferase (ALT), creatinine (CRE), and blood urea nitrogen (BUN) for liver and kidney function assessment. Major organs (heart, liver, spleen, lungs, and kidneys) were fixed in 10% PFA and examined by hematoxylin and eosin (H&E) staining for histopathological evaluation.

### Construction of the NAFLD-HCC mouse model

The mouse model was prepared as previously reported method [45]. Before model induction, mice received tail vein injections of lentiviruses carrying sh-NC or sh-GPR43 (RIBOBIO, China; 100 µL, 1 × 10⁸ PFU/mL). The target sequences are listed in Table S1. A single intraperitoneal injection of diethylnitrosamine (DEN, 25 mg/kg; N0756, Sigma) was administered to C57BL/6J mice. At 6 weeks of age, the normal chow was replaced with a high-fat/high-cholesterol diet (HFHC; SF11-078, Specialty Feeds, WA). Mice were divided into the following groups: Normal (standard diet, untreated), Model (DEN + HFHC, gavage with distilled water), CS/PT-NPs (DEN + HFHC, 50 mg/kg/day CS/PT-NPs), APs-CS/PT-NPs (DEN + HFHC, 50 mg/kg/day APs-CS/PT-NPs), APs-S (DEN + HFHC, 50 mg/kg/day APs solution), APs-CS/PT-NPs + sh-NC (sh-NC lentivirus + DEN + HFHC + 50 mg/kg/day APs-CS/PT-NPs), and APs-CS/PT-NPs + sh-GPR43 (sh-GPR43 lentivirus + DEN + HFHC + 50 mg/kg/day APs-CS/PT-NPs). For acetate-treated HCC mice, a HFHC diet supplemented with 0.4% sodium acetate (S8750, Sigma) was provided from 6 weeks of age, while controls received HFHC diet with distilled water (Vehicle and Acetate groups). At 26 weeks of age, mice were euthanized, and serum, liver, intestinal, and fecal samples were collected for analysis [46]. The therapeutic dose of 50 mg/kg/day was selected based on previously reported optimal efficacy [46] and validated by preliminary dose-optimization experiments.

### NAFLD–HCC organoid culture

Liver organoids from NAFLD–HCC mice were established following the method of Xiaofang Huang et al. [45] with slight modifications. Under sterile conditions, livers were dissected, rinsed with cold Hank’s balanced salt solution (HBSS) containing antibiotics, and mechanically dissociated into cell clusters. The resulting suspension was embedded in Matrigel (356234, Corning, USA) and cultured in DMEM/F12 medium (11320033, Thermo Fisher, USA) supplemented with 100 U/mL penicillin–streptomycin, 10 mM HEPES (15630106, Thermo Fisher), GlutaMAX (35050061, Thermo Fisher), B27 (C0347, Beyotime), N2 (17502048, Thermo Fisher), 30% Wnt3a, 10% R-spondin, 10% murine Noggin (conditioned media), 10 µM forskolin (F6886, Sigma), 100 ng/mL murine EGF (SRP3196, Sigma), 100 ng/mL FGF10 (GF172, Sigma), 25 ng/mL HGF (SRP6014, Sigma), 10 nM gastrin I (HY-P1097, MCE), 1.25 mM N-acetylcysteine (50303ES05, Yeasen, China), 10 mM nicotinamide (72340, Sigma), 5 µM A8301 (53002ES03, Yeasen), and 10 µM Y27632 (HY-10071, MCE). Organoids developed into spheroid or glandular structures within 3–7 days and were co-cultured with sodium acetate for 6 days before collection for subsequent experiments.

### RNA extraction and RT-qPCR

RNA extraction and RT-qPCR were performed following the method of Shuai Ye et al. [47] with modifications. Total RNA was extracted from samples using TRIzol reagent (Takara, Japan). For each sample, 1 µg total RNA was reverse transcribed using the PrimeScript RT Reagent Kit with gDNA Eraser (Takara, Japan). Quantitative PCR was performed using SYBR Premix Ex Taq II (Takara, Japan) on a LightCycler 480 system (Roche, USA). The amplification protocol consisted of one cycle of 95 °C for 5 min, followed by 40 cycles of 95 °C for 10 s and 60 °C for 30 s, and a final melting curve step (95 °C for 15 s, 60 °C for 1 min, and 95 °C for 15 s). GAPDH served as the internal control, and relative gene expression was calculated using the 2^⁻ΔΔCt^ method. Primers were synthesized by Qingke Biological Technology (Shanghai, China) and were as follows: GAPDH-F: CCCTTAAGAGGGATGCTGCC; GAPDH-R: TACGGCCAAATCCGTTCACA; GPR43-F: CTTGATCCTCACGGCCTACAT; GPR43-R: CCAGGGTCAGATTAAGCAGGAG.

### Germ-free mouse model

Germ-free C57BL/6J mice were orally gavaged three times per week with *B. pseudolongum* and daily gavaged with CS/PT-NPs or APs-CS/PT-NPs (50 mg/kg/day). Mice were divided into two groups: CS/PT-NPs and APs-CS/PT-NPs. After one month of treatment, the animals were euthanized, and portal vein blood was collected for metabolomic profiling. Portal blood, fecal samples, and liver tissues were also obtained for acetate quantification. Acetate was derivatized with 3-nitrophenylhydrazine and analyzed using liquid chromatography–mass spectrometry (LC-MS) under negative ion multiple reaction monitoring [46].

### Subcutaneous xenograft mouse model

A human HCC xenograft mouse model was established using Huh-7 cells. Huh-7 cells (1 × 10⁷ in 0.1 mL ice-cold PBS) were subcutaneously injected into the right dorsal side of 4-week-old nude mice. Five weeks post-injection, mice were randomly divided into two groups: Acetate (200 mg/kg/day, oral gavage) and Vehicle (distilled water, oral gavage). Tumor volume was recorded weekly, and at the end of the study, mice were euthanized and tumors were excised and weighed [46].

### Intestinal permeability measurement

Intestinal permeability was assessed using the FITC–dextran assay, following the method of Yuge Zhao et al. [48] with minor modifications. After 3 h of fasting, mice were orally gavaged with 150 µL of FITC–dextran (4 kDa, 80 mg/mL; MSO901, Shanghai Maokang Biotechnology Co., Ltd.). Four hours later, blood was collected from the tail vein and centrifuged at 10,000 g for 10 min at ambient temperature. Serum was diluted with PBS (1:4), and fluorescence intensity was measured using a fluorescence spectrophotometer (excitation: 485 nm; emission: 528 nm). FITC–dextran concentrations were determined from a standard curve prepared by serial dilution of the gavage solution in PBS.

### Histological analysis

Liver tissue was fixed in a 4% PFA solution for 24 h. Following fixation, the tissue underwent a graded ethanol dehydration, clearing, and routine paraffin embedding procedure. Section (3 μm) were cut using a microtome, baked at 60 °C for 1 h, and dewaxed with xylene. After rehydration, H&E staining was performed using a commercial kit (C0105S, Beyotime). Sections were stained with hematoxylin for 2 min, differentiated in 1% hydrochloric acid–ethanol for 10 s, and counterstained with eosin for 1 min. Slides were dehydrated, cleared, and mounted in neutral resin. Morphological changes were examined under an optical microscope (IX53, Thermo Fisher), and two blinded investigators independently evaluated hepatic steatosis and inflammation. Fat degeneration scores were graded as 0 (< 5%), 1 (5%–33%), 2 (33%–66%), and 3 (>66%), while inflammation scores were defined as 0 (none), 1 (< 2 foci/200×), 2 (2–4 foci/200×), and 3 (>4 foci/200×) [49].

Masson trichrome (G1340, Solarbio) and Sirius Red (G1472, Solarbio) staining were used to evaluate collagen deposition. Images were captured under an optical microscope and quantified using ImageJ [50].

For lipid accumulation, frozen liver Sects. (5–8 μm) were fixed in 4% PFA for 10–15 min, rinsed with distilled water, and stained with freshly prepared Oil Red O for 8–10 min. Sections were briefly differentiated in 75% ethanol for 5–10 s, counterstained with hematoxylin for 1 min, and mounted with aqueous medium. Lipid droplets appeared bright red, nuclei blue, and background nearly unstained. Quantification was performed using ImageJ [50].

### Immunohistochemistry (IHC)

Expression of Claudin-1 and E-Cadherin in colon tissue and Ki-67 in liver tissue was analyzed using paraffin-embedded sections. After deparaffinization and antigen retrieval in citrate buffer, sections were treated with 1% hydrogen peroxide to block endogenous peroxidase activity and then incubated with 5% BSA for blocking. Primary antibodies against Ki-67 (ab15580, 1 µg/mL), Claudin-1 (ab307692, 1:100), and E-Cadherin (ab314063, 1:400, all from Abcam, UK) were applied overnight at 4 °C. After three washes with PBS, sections were incubated with an HRP-conjugated secondary antibody (Goat anti-Rabbit IgG H&L, ab6721, 1:1000, Abcam, UK), followed by DAB color development. Images were captured using an optical microscope (IX53, Thermo Fisher). The staining intensity and area were quantified with ImageJ software. Mean optical density (OD) was calculated using formula (1), and the percentage of positive cells (dark brown) was determined using formula (2) [51].

Formula (1): $$\:\text{OD}=-log10(\frac{\text{Transmitted light intensity}}{\text{Incident light intensity}})$$

Formula (2): $$\:\text{Percentage of positive cells}\:(\%)=\frac{\text{Number of positive cells}}{\text{Total cell count}}\times100$$

### Intraperitoneal glucose tolerance test (IPGTT) and insulin tolerance test (ITT) tests

Following an overnight fast, mice received intraperitoneal injections of glucose (1 g/kg; PHR1000, Sigma) for IPGTT or insulin (0.5 U/kg; Y0001717, Sigma) for ITT. Blood glucose levels were recorded every 30 min, and the area under the curve (AUC) was calculated using GraphPad Prism software [52].

### Biochemical assays

Serum alpha-fetoprotein (AFP) was quantified using a Mouse AFP ELISA Kit (PA016, Beyotime). Hepatic IL-6, IL-1β, and TNF-α levels were measured with ELISA kits (PI326, PI301, PT512; Beyotime). Serum ALT, AST, and triglycerides (TG) were analyzed using a Catalyst One chemical analyzer (IDEXX Laboratories). Portal vein LPS concentrations were determined using a Mouse LPS ELISA Kit (CSB-E13066m, CUSABIO).

### Acetate pull-down assay

Recombinant G protein–coupled receptor 43 (GPR43; CSB-YP008605MO1, Huamei Biotechnology, China) was incubated with sodium acetate and either anti-GPR43 antibody (84544-1-RR, 1:1000, Proteintech) or normal rabbit IgG (ab172730, 1:1000, Abcam, UK) in Tris buffer (pH 7.4) overnight at 4 °C. The mixture was then incubated with Dynabeads™ Protein G (Invitrogen, USA) for 2 h at ambient temperature. After washing, bound complexes were eluted with 50 mM glycine (pH 2.8). Eluates were analyzed by WB to confirm GPR43 binding, and the remaining fraction was examined by LC–MS to detect acetate.

### Cell Immunofluorescence

Cells were washed twice with PBS to remove residual culture medium and fixed with 4% PFA for 30 min. After permeabilization with 0.3% Triton X-100 for 15 min, cells were blocked with 5% bovine serum albumin (BSA) in PBS for 1 h at 37 °C to prevent nonspecific binding. Subsequently, cells were incubated overnight at 4 °C with an anti-Ki-67 primary antibody (ab15580, 1 µg/mL, Abcam, UK). After three PBS washes, cells were incubated with goat anti-rabbit IgG H&L (Alexa Fluor^®^ 488) secondary antibody (ab150077, 1:1000, Abcam, UK) for 1 h at 37 °C in the dark, followed by DAPI staining. Images were acquired using a confocal laser scanning microscope, and fluorescence intensity–based protein quantification was performed using ImageJ software (Rawak Software, Germany).

### WB

Total proteins from liver tissue and cells were extracted using Rapid Tissue (EX2410, Solarbio, China) or Fixed Cell (EX2170, Solarbio, China) Protein Extraction Kits. Protein concentration was determined with the BCA kit (BCA1-1KT, Sigma, USA). Equal protein amounts (20 µg) were separated by 10–12% SDS–PAGE, transferred onto PVDF membranes (Millipore, USA), and blocked with 5% BSA for 2 h. Membranes were incubated overnight at 4 °C with primary antibodies (Table S2), followed by HRP-conjugated goat anti-rabbit IgG (ab6721, 1:2000, Abcam, UK) for 2 h. Bands were visualized using enhanced chemiluminescence (iBright FL1500, Thermo Fisher, USA). β-Actin (ab8226, 1 µg/mL, Abcam, UK) served as a loading control. All experiments were performed in triplicate.

### 16 S rRNA amplicon sequencing and analysis

Fecal samples from the APs-CS/PT-NPs and CS/PT-NPs groups were collected, and bacterial DNA was extracted using the Stool Genomic DNA Kit (QIAGEN, Germany). The V3–V4 region of the bacterial 16 S rRNA gene was amplified by qPCR with primers 338 F (5′-ACTCCTACGGGAGGCAGCAG-3′) and 806R (5′-GGACTACHVGGGTWTCTAAT-3′). Sequencing was performed on an Illumina HiSeq 2500 platform using paired-end 250 bp reads. High-quality reads were processed using the QIIME software package. Alpha diversity indices (Richness, Chao1, ACE) were used to evaluate microbial richness and evenness, while Beta diversity was analyzed via Constrained Principal Coordinates Analysis (CPCoA). Group differences in microbial abundance and diversity were assessed using the Wilcoxon rank-sum and Welch’s t-tests. Differentially abundant taxa were identified using the edgeR package in R, and results were visualized with volcano plots and Linear Discriminant Analysis Effect Size (LEfSe). Linear Discriminant Analysis (LDA) scores indicated the relative impact of taxa contributing to differences between groups.

### Targeted metabolomics

Sterile portal vein blood samples (50 µL) were collected from mice for targeted metabolomic analysis of SCFAs, conducted by Novogene. Samples were diluted 1:10 (w/v) and mixed with an equal volume of 50% aqueous acetonitrile for protein precipitation. After thorough mixing, samples were centrifuged (10,000 g, 10 min, 4 °C), and the resulting supernatant was analyzed for SCFAs using liquid chromatography–mass spectrometry (LC–MS) and gas chromatography–mass spectrometry (GC–MS).

### Quality control of sequencing data and reference genome alignment

Liver tissues from mice in the APs-CS/PT-NPs and CS/PT-NPs groups (*n* = 3) were collected, and total RNA was extracted using TRIzol reagent (15596026, Thermo Fisher Scientific). RNA purity was assessed using a Nanodrop ND-1000 spectrophotometer based on OD260/280 ratios, and RNA concentration was quantified with the Qubit RNA Assay Kit (N608303-0100, Sangon Biotech). Samples meeting the criteria of RNA Integrity Number (RIN) ≥ 7.0 and 28 S:18 S ratio ≥ 1.5 were used for library construction.

For each sample, 5 µg of RNA was processed. Ribosomal RNA was removed using the Ribo-Zero™ Magnetic Kit (MRZH116, Epicentre Technologies, USA). Libraries were prepared using the NEBNext Ultra RNA Library Prep Kit (E7770, NEB, USA). RNA was fragmented into ~ 300 bp fragments, followed by first- and second-strand cDNA synthesis. End repair, A-tailing, and adapter ligation were performed, and the second cDNA strand was selectively digested using USER Enzyme (M5505S, NEB, USA) to generate strand-specific libraries. The libraries were PCR-amplified, purified, validated with the Agilent 2100 Bioanalyzer, and quantified using the KAPA Library Quantification Kit (TAQDKB, KAPA Biosystems). Sequencing was carried out on an Illumina NextSeq CN500 platform using paired-end reads.

Raw reads were processed with Trimmomatic for quality control, including adapter removal, trimming of low-quality bases, and filtering of low-quality reads. Quality metrics such as read counts, Q30 scores, and GC content were calculated. Clean reads were then aligned to the NCBI mouse reference genome using HISAT2.

### Differential gene expression analysis

Differential expression analysis between the APs-CS/PT-NPs and CS/PT-NPs groups was performed using the “edgeR” package in R, based on the read count data for lncRNA and mRNA. A |log_2_FC| >1 and *p*-value < 0.05 were used as the criteria for selecting differentially expressed genes (DEGs). The “ggplot2” package in R was used to generate volcano plots, while the “heatmap” package in R was used to create heatmaps of the DEGs.

### Gene functional enrichment analysis

Gene Ontology (GO) and Kyoto Encyclopedia of Genes and Genomes (KEGG) enrichment analyses of mRNA were performed using the “clusterProfiler” package in R. Bubble plots were generated using the “enrichplot” package to visualize the enrichment results of three GO categories: Biological Process (BP), Cellular Component (CC), and Molecular Function (MF), as well as the KEGG enrichment results.

### Statistical analysis

All data were analyzed using GraphPad Prism software (version 9.5.0). Continuous variables were expressed as mean ± standard deviation (SD). An independent sample t-test was used for comparisons between two groups, while a one-way analysis of variance (ANOVA) was applied for comparisons among multiple groups, followed by Tukey’s post hoc test. A *p* value of < 0.05 was considered statistically significant.

## Results

### Preparation and characterization of APs-CS/PT-NPs

**Fig. 1:**
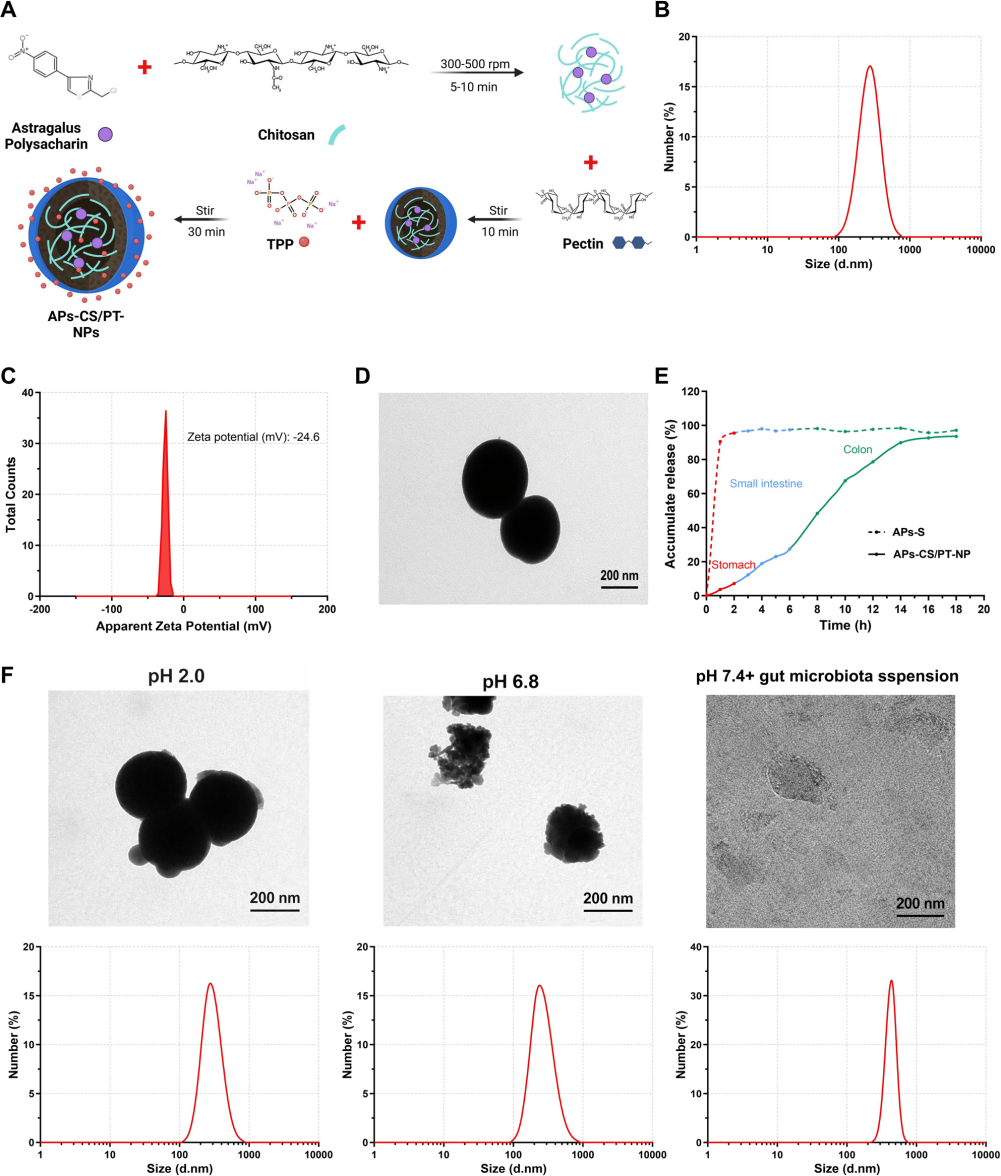
Characterization and in vitro release profile of APs-CS/PT-NPs. Note: (**A**) Schematic illustration of APs-CS/PT-NPs preparation; (**B**) Representative particle size distribution of APs-CS/PT-NPs; (**C**) Zeta potential of APs-CS/PT-NPs; (**D**) Representative morphology of APs-CS/PT-NPs (scale bar: 200 nm); (**E**) Release profile of APs from APs-CS/PT-NPs under different physiological pH conditions, assessed using the dialysis method, with APs solution (APs-S) as a control; (**F**) TEM images of APs-CS/PT-NPs under different physiological pH conditions (scale bar: 200 μm)

APs containing negatively charged carboxyl groups were electrostatically complexed with positively charged CS and negatively charged PT to form stable NPs (Fig. 1A). DLS analysis revealed that the APs-CS/PT-NPs had an average particle size of 276.3 ± 12.7 nm (Fig. 1B) with a PDI of 0.17 ± 0.01, indicating a uniform particle distribution suitable for nanodrug delivery systems. The zeta potential was − 24.6 ± 2.3 mV (Fig. 1C), suggesting strong surface charge repulsion that contributed to excellent colloidal stability. TEM imaging confirmed spherical morphology and uniform size, consistent with the DLS findings (Fig. 1D). Furthermore, the EE was 94.6%, and the drug loading capacity reached 14.3%, demonstrating the successful fabrication of APs-CS/PT-NPs with high drug-loading efficiency and stability by optimizing electrostatic interactions and crosslinking conditions. After 35 days of storage at 4 °C, APs-CS/PT-NPs exhibited negligible changes in particle size, zeta potential, or morphology, confirming robust long-term stability (Figure S1A–C). FTIR spectra showed significant alterations in carboxyl–amino regions compared with individual CS and PT molecules, without new absorption peaks, indicating that nanoparticle formation occurred via electrostatic interactions between –NH₃⁺ groups of CS and –COO⁻ groups of PT (Figure S1D). Electrostatic attraction promoted nanoparticle assembly, while electrostatic repulsion maintained dispersion stability. Hydrogen bonding further contributed to structural organization and network stability, collectively influencing nanoparticle formation and long-term stability.

We evaluated the sustained-release behavior of APs-CS/PT-NPs in different simulated gastrointestinal environments. After oral administration, the formulation sequentially passes through the stomach (pH 1.5–3.5, 1–2 h), small intestine (pH 5.5–6.8, 3–6 h), and colon (pH 6.4–7.8, rich in microbiota, 12–24 h). To mimic these physiological conditions, drug release was tested in simulated gastric fluid (SGF, pH 2.0), simulated intestinal fluid (SIF, pH 6.8), and simulated colonic fluid (SCF, pH 7.4, containing 20% murine cecal filtrate). No burst release occurred in SGF or SIF, indicating that strong electrostatic interactions between negatively charged APs and the CS/PT matrix effectively restricted premature drug release. As shown in Fig. 1E, only 6.8% of APs were released after 2 h in SGF, increasing to 23.7% after 4 h in SIF. In contrast, incubation in SCF resulted in 85.1% release within 14 h, confirming the colonic responsiveness of the formulation. TEM images and size distribution analysis of APs-CS/PT-NPs in different simulated biological fluids (Fig. 1F) confirmed their pH- and microbiota-responsive characteristics. In the lower gastrointestinal tract, deprotonation of CS weakens its electrostatic interactions with PT compared to the upper tract, while GM facilitate the enzymatic degradation of both CS and PT. The synergistic effects of microbial degradation and reduced electrostatic attraction result in nanoparticle disintegration and a rapid release of APs in the colon. These findings suggest that CS/PT-NPs hold promise as a colon-targeted delivery system for APs. The unique properties of APs-CS/PT-NPs may help protect the active drug from enzymatic degradation in the intestine and minimize interactions with the small intestinal mucosa, potentially enhancing therapeutic efficacy while reducing irritation to the upper gastrointestinal tract.

To evaluate the clinical applicability of APs-CS/PT-NPs, we conducted a long-term safety assessment in mice over three months, examining the biocompatibility of each group. H&E staining showed no noticeable histopathological abnormalities in major organs between treated and untreated groups (Figure S2A). Serum biochemical parameters, including ALT, AST, CRE, and BUN, also remained unchanged among all groups (Figure S2B). These results demonstrate that the APs-CS/PT-NP system possesses excellent biosafety and long-term biocompatibility.

### APs-CS/PT-NPs attenuate HFHC-Induced liver injury and lipid Accumulation, inhibiting HCC development

**Fig. 2:**
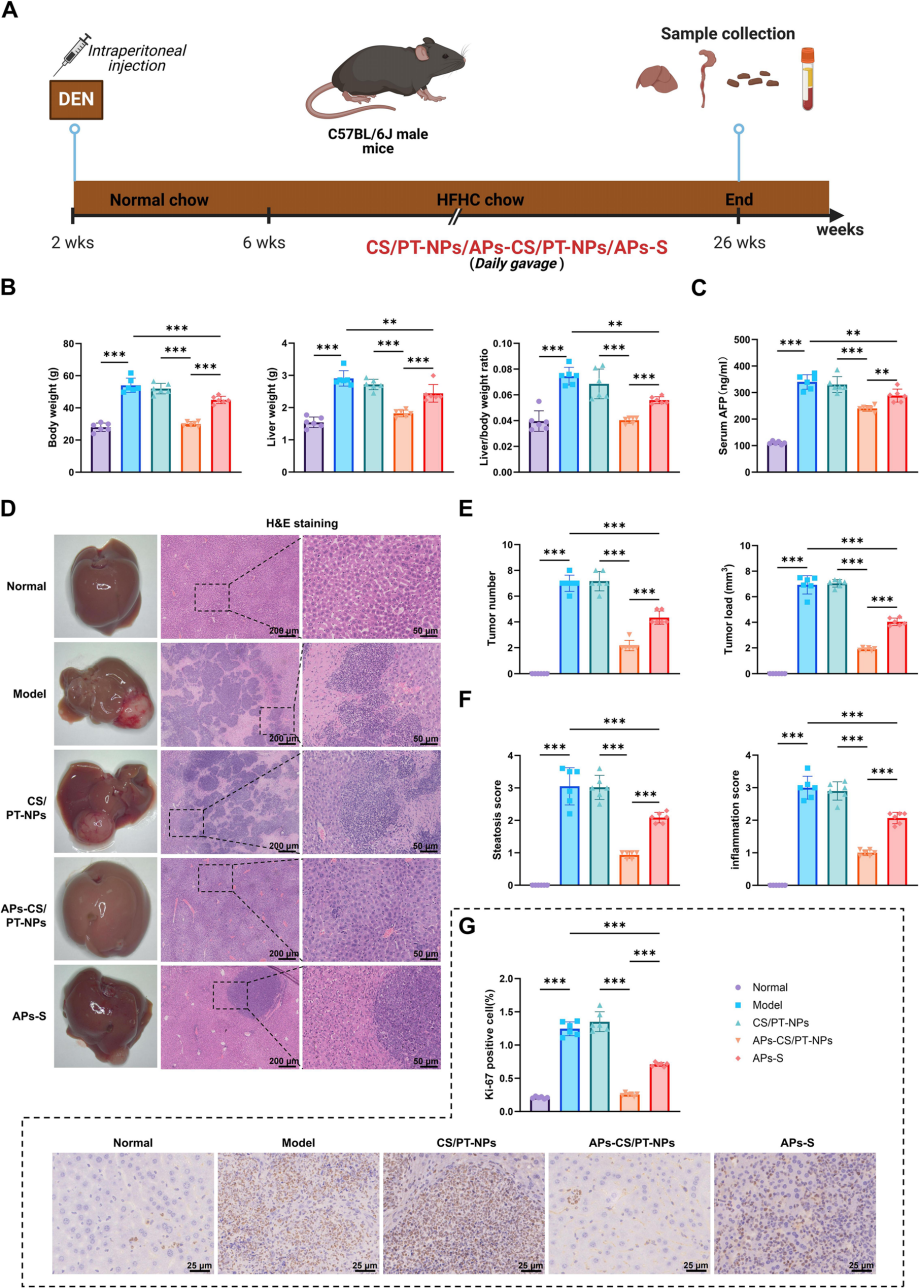
Effects of APs-CS/PT-NPs on Tumor Formation in an HCC Mouse Model. Note: (**A**) Experimental design illustrating dietary treatment and oral administration of APs-CS/PT-NPs in the HCC mouse model; (**B**) Statistical analysis of body weight, liver weight, and liver-to-body weight ratio in different groups; (**C**) Serum AFP levels in each group; (**D**) Gross morphology of the liver and representative H&E-stained liver tissue sections (scale bar: 200 μm; magnified view scale bar: 50 μm); (**E**) Tumor count and tumor burden analysis in different groups; (**F**) Hepatic steatosis and inflammation scores in each group; (**G**) Immunohistochemical staining of Ki-67 expression in liver tissues (scale bar: 25 μm). *p < 0.05, **p < 0.01, ***p < 0.001; *N* = 6

To evaluate the therapeutic efficacy of APs-CS/PT-NPs against HCC progression, a NAFLD–HCC mouse model was established via a single intraperitoneal injection of N-diethylnitrosamine (DEN) followed by a high-fat, high-cholesterol (HFHC) diet. Mice received daily oral gavage of APs solution (APs-S), CS/PT-NPs, or APs-CS/PT-NPs (Fig. 2A). At 26 weeks, mice were euthanized for analysis. Compared with the Normal group, the Model group showed significant increases in body weight, liver weight, liver-to-body weight ratio, and serum AFP levels. No differences were found between the CS/PT-NPs and Model groups. However, both the APs-S and APs-CS/PT-NPs groups exhibited significant reductions in these parameters, with the APs-CS/PT-NPs group showing the strongest effects (Fig. 2B-C). At 26 weeks, mice were euthanized for analysis. Compared with the Normal group, the Model group showed significant increases in body weight, liver weight, liver-to-body weight ratio, and serum AFP levels. No differences were found between the CS/PT-NPs and Model groups. However, both the APs-S and APs-CS/PT-NPs groups exhibited significant reductions in these parameters, with the APs-CS/PT-NPs group showing the strongest effects (Fig. 2D-F). Immunohistochemical staining of Ki-67 revealed markedly increased hepatocyte proliferation in the Model group, whereas both APs-S and APs-CS/PT-NPs treatment significantly inhibited Ki-67 expression, with the latter exhibiting the greatest suppression (Fig. 2G).

Biochemical analyses further supported these findings. The Model group showed elevated serum ALT, AST, and hepatic TG levels compared with the Normal group, while APs-S and APs-CS/PT-NPs treatment significantly reduced these indicators, with APs-CS/PT-NPs showing the strongest effect (Figure S3A). Oil Red O staining confirmed extensive lipid accumulation in the Model group, which was markedly reduced following treatment, especially with APs-CS/PT-NPs (Figure S3B-C). Additionally, APs-CS/PT-NPs significantly improved glucose tolerance by enhancing insulin sensitivity, thereby lowering blood glucose levels (Figure S3D-E).

These findings suggest that APs-CS/PT-NPs alleviate hepatic injury and lipid accumulation induced by a high-fat, high-cholesterol diet, ultimately inhibiting HCC development.

### APs-CS/PT-NPs modulate GM and improve intestinal barrier function

**Fig. 3:**
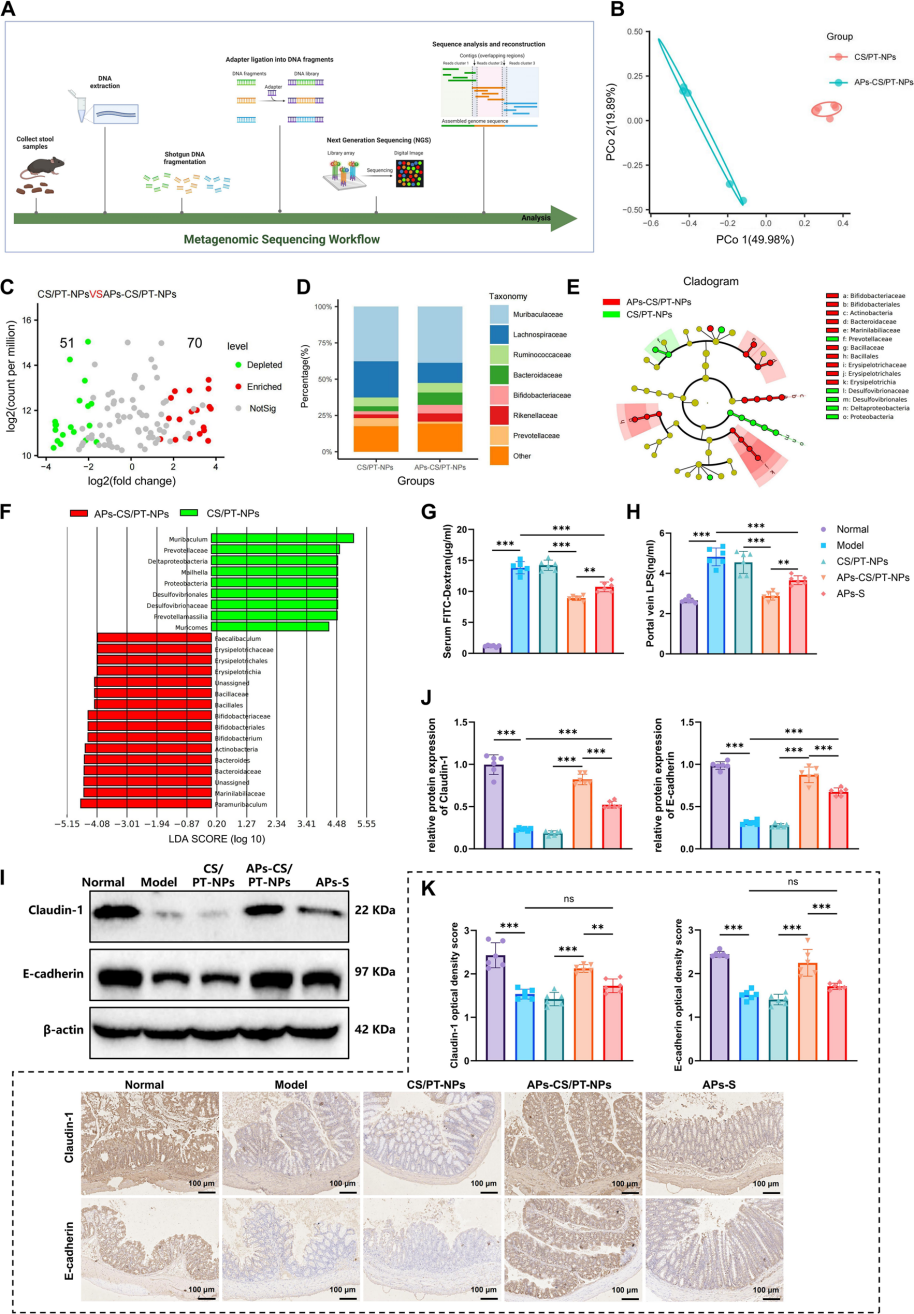
Impact of APs-CS/PT-NPs on the Gut Microbiota and Intestinal Barrier Function in HCC Model Mice. Note: **A** Workflow diagram for analyzing the composition of gut microbiota in fecal samples from HCC model mice using 16 S sequencing (*N* = 5). **B** Beta diversity analysis of the gut microbiota in mice treated with APs-CS/PT-NPs compared to CS/PT-NPs. **C** Volcano plot comparing the abundance differences between the APs-CS/PT-NPs and CS/PT-NPs groups; red dots indicate enriched ASVs, green dots indicate depleted ASVs, and grey dots represent non-significant differences, with numbers indicating the quantity of differentially enriched ASVs. **D** Stacked bar chart showing the relative abundance of microbial taxa at the family level, categorized by treatment group. **E** Cladogram illustrating the phylogenetic distribution of microbial species abundance differences between the APs-CS/PT-NPs and CS/PT-NPs groups. **F** Bar chart of LDA scores depicting species abundance in the gut microbiota between the APs-CS/PT-NPs and CS/PT-NPs groups. **G** FITC-dextran assay results for serum intestinal permeability in each group. **H** ELISA results for LPS levels in portal venous blood of mice from each group. **I**-**J** WB analyses of Claudin-1 and E-cadherin protein expression levels in colon tissue from each group. **K** Immunohistochemical analysis of Claudin-1 and E-cadherin expression in colon tissues (scale bar: 100 μm); * indicates *p* < 0.05, **p < 0.01, ***p < 0.001 compared between groups; *N* = 6

To investigate the effects of APs-CS/PT-NPs on GM composition, we performed 16 S rRNA sequencing on fecal samples from HCC mice in both the APs-CS/PT-NPs and CS/PT-NPs groups (Fig. 3A). Alpha diversity indices, including Richness, Chao1, and ACE, as well as rarefaction curves, differed significantly between the two groups (Figure S4). Beta diversity analysis further revealed clear group separation (Fig. 3B), suggesting consistent microbial compositional differences.

To further characterize microbial compositional differences between the APs-CS/PT-NPs and CS/PT-NPs groups, taxonomic profiling was performed at the family level. The analysis revealed significant variations in amplicon sequence variants (ASVs), with 51 ASVs significantly decreased and 70 ASVs significantly increased in the CS/PT-NPs group compared with the APs-CS/PT-NPs group (Fig. 3C). Consistent with these findings, stacked bar plots of relative abundance at the family level demonstrated distinct microbial community structures between groups, with a pronounced enrichment of *Bifidobacteriaceae* in the APs-CS/PT-NPs group (Fig. 3D).

LEfSe analysis was conducted to identify bacterial taxa that differed significantly between the APs-CS/PT-NPs and CS/PT-NPs groups. The relative abundances of *Muribaculaceae*, *Prevotellaceae*, *Prevotellamassilia*, *Mailhella*, *Desulfovibrionaceae*, *Proteobacteria*, and *Deltaproteobacteria* were markedly higher in the CS/PT-NPs group, indicating enrichment of pro-inflammatory taxa. In contrast, the APs-CS/PT-NPs group showed significant enrichment of *Bacteroides*, *Paramuribaculum*, *Actinobacteria*, *Marinilabiliaceae*, *Bifidobacterium*, *Faecalibaculum*, *Bacillales*, and *Erysipelotrichales* (Fig. 3E-F), suggesting that APs-CS/PT-NPs promote the proliferation of beneficial bacteria involved in maintaining intestinal homeostasis. Similarly, previous evidence has demonstrated that APs can modulate GM, enhance intestinal barrier function, and improve immune responses, thereby alleviating conditions such as colitis, diabetes, and fatty liver disease [53–56].

Given that HCC is closely linked to gut barrier dysfunction and microbial translocation through the gut–liver axis [57–59], we further investigated the effects of APs-CS/PT-NPs on intestinal barrier function in HCC mice. Compared with the Normal group, the Model group exhibited markedly elevated serum FITC-dextran levels and increased LPS concentrations in portal vein blood, indicating enhanced intestinal permeability. In contrast, treatment with APs-S or APs-CS/PT-NPs reduced both indicators, with APs-CS/PT-NPs showing the strongest protective effect (Fig. 3G-H). WB and immunohistochemical analyses revealed significant downregulation of the tight junction proteins Claudin-1 and E-cadherin in the Model group compared with the Normal group. Both APs-S and APs-CS/PT-NPs treatments restored the expression of these proteins, with the APs-CS/PT-NPs group displaying the most robust improvement (Fig. 3I-K).

These findings suggest that APs-CS/PT-NPs can modulate GM and enhance intestinal barrier function, thereby mitigating gut-liver axis dysregulation and inhibiting HCC progression.

### APs-CS/PT-NPs enhance the abundance of *Bifidobacterium pseudocatenulatum* and promote acetate production

**Fig. 4:**
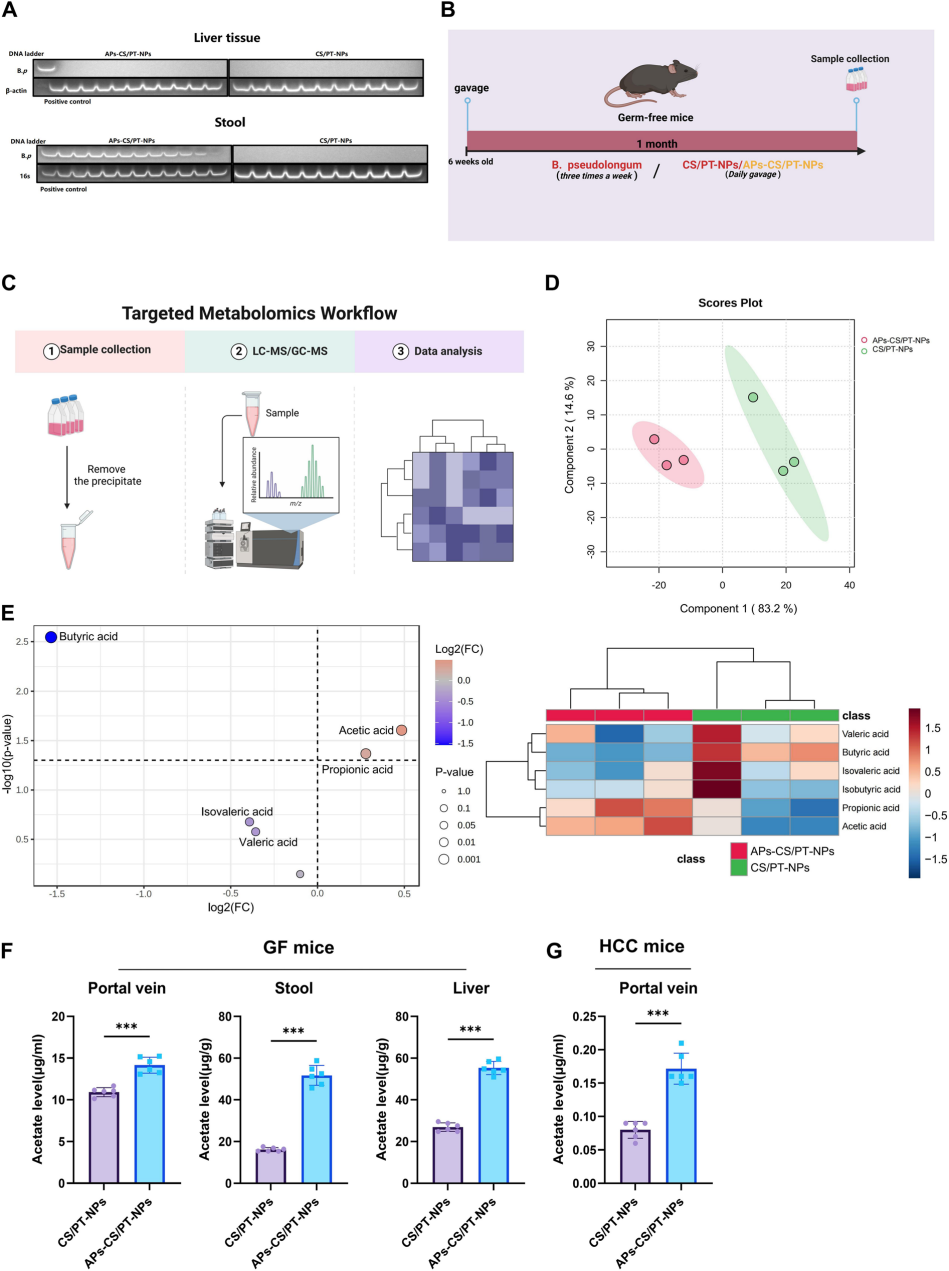
Investigation of Metabolic Regulation by APs-CS/PT-NPs in the HCC Mouse Model. Note: (**A**) Relative abundance of Bifidobacterium pseudolongum in fecal and liver tissue samples from different groups, analyzed via PCR; (**B**) Schematic diagram of the experimental procedure in germ-free mice; (**C**) Workflow of targeted SCFA metabolomics analysis; (**D**) PLS-DA plot comparing different groups in targeted SCFA metabolomics analysis; (**E**) Volcano plot and heatmap illustrating differential SCFA metabolites in the portal vein of germ-free mice; (**F**) Acetate levels in feces, portal vein blood, and liver tissues of germ-free mice; (**G**) Acetate levels in the portal vein blood of HCC model mice. ***p < 0.001 compared between groups; *N* = 6

Previous research has demonstrated that *Bifidobacterium pseudocatenulatum* exerts antitumor and immunoregulatory effects in HCC, colorectal cancer, and melanoma [46, 60, 61]. In our 16 S rRNA sequencing analysis, APs-CS/PT-NPs treatment significantly increased the abundance of *Bifidobacterium* (Fig. 3C), suggesting a potential link between microbial modulation and the therapeutic efficacy of APs-CS/PT-NPs. Since *B. pseudocatenulatum* is a key species within this genus, its distribution was further examined. Although undetectable in hepatic tissues of treated HCC mice, *B. pseudocatenulatum* was abundant in fecal samples (Fig. 4A), implying that its bioactive metabolites, rather than bacterial translocation, contribute to the observed antitumor effects.

To further investigate this, we conducted targeted SCFA metabolomic analysis on portal vein blood samples from germ-free mice administered *B. pseudocatenulatum* in combination with either APs-CS/PT-NPs or CS/PT-NPs for one month (Fig. 4B-C). The metabolomic analysis revealed distinct SCFA profiles between the two groups (Fig. 4D), with a significant elevation of acetic acid in the APs-CS/PT-NPs group (Fig. 4E). Since acetic acid and acetate exist in a dynamic equilibrium under physiological conditions, acetate levels were further quantified. Acetate concentrations were markedly increased in feces, portal vein blood, and liver tissue of germ-free mice treated with APs-CS/PT-NPs (Fig. 4F), as well as in the portal vein serum of HCC mice (Fig. 4G). Previous studies have demonstrated that *B. pseudocatenulatum* produces SCFAs [46, 62]. Furthermore, APs have been shown to alleviate hepatic steatosis by increasing SCFA levels, particularly acetate, and regulating hepatic lipid metabolism in mice [63], which aligns with our findings.

In conclusion, our results indicate that APs-CS/PT-NPs enhance the abundance of *B. pseudocatenulatum*, leading to increased production of acetic acid and acetate.

### Acetate inhibits HCC both in vitro and in vivo

**Fig. 5:**
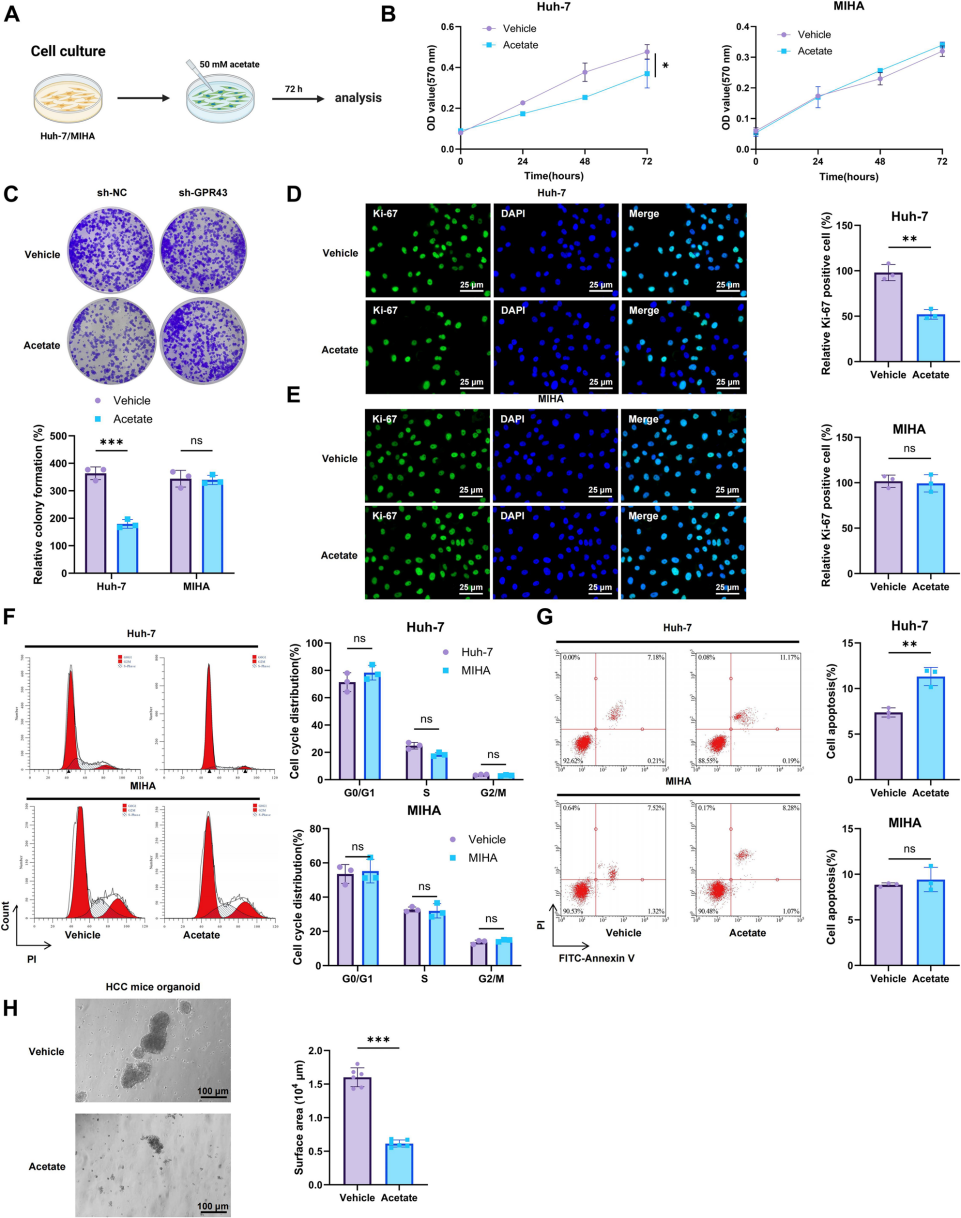
Antitumor Effects of Acetate on the HCC Cell Line Huh-7. Note: (**A**) Schematic representation of Huh-7 and MIHA cell treatments with Acetate or distilled water; (**B**) MTT assay assessing the viability of Huh-7 and MIHA cells in different treatment groups; (**C**) Colony formation assay evaluating the clonogenic capacity of Huh-7 and MIHA cells; (**D**–**E**) Immunofluorescence analysis of Ki-67-positive cells in Huh-7 and MIHA cells, with quantification (scale bar: 25 μm); (**F**–**G**) Flow cytometry analysis of the cell cycle distribution and apoptosis in Huh-7 cells; (**H**) Organoid culture assay assessing the inhibitory effects of Acetate on the growth of mouse HCC organoids (scale bar: 100 μm). *p < 0.05, **p < 0.01, ***p < 0.001 compared to the control group; *N* = 6; in vitro experiments were performed in triplicate

To investigate the effects of acetate on HCC, we treated human HCC cells (Huh-7) and normal liver cells (MIHA) with acetate, using distilled water as a control (Fig. 5A). MTT and colony formation assays showed that acetate markedly reduced the viability and colony-forming ability of Huh-7 cells while exerting no cytotoxic effect on MIHA cells (Fig. 5B-C). Immunofluorescence staining demonstrated a pronounced decrease in Ki-67–positive Huh-7 cells after acetate treatment, indicating reduced proliferation (Fig. 5D-E). Flow cytometry further revealed that acetate induced apoptosis and caused G1/S phase arrest in Huh-7 cells (Fig. 5F-G). Wound healing and Transwell assays further confirmed that acetate impaired the migratory capacity of Huh-7 cells (Figure S5A-B). In addition, acetate treatment downregulated the expression of proliferating cell nuclear antigen (PCNA), cyclin D1, and vimentin, while promoting the cleavage of caspase-7 and PARP in Huh-7 cells (Figure S5C). Finally, acetate significantly inhibited the growth of HCC organoids in a mouse model (Fig. 5H).

We established a subcutaneous xenograft model in nude mice by injecting Huh-7 cells, followed by acetate treatment (Figure S6A). After six weeks, tumor volume and weight were markedly reduced in acetate-treated mice compared with controls (Figure S6B-C). In the DEN + HFHC diet–induced HCC model (Figure S6D), acetate treatment significantly reduced body weight, liver weight, liver-to-body weight ratio, and serum AFP levels (Figure S6E-F). Tumor burden was markedly lower, and liver damage was alleviated (Figure S6G-I). Ki-67 immunostaining showed a pronounced reduction in hepatocyte proliferation in the acetate-treated group (Figure S6J). Furthermore, acetate treatment significantly decreased hepatic TG levels, serum ALT, and AST levels (Figure S7A), and hepatic lipid droplet accumulation (Figure S7B), while improving glucose tolerance and insulin sensitivity (Figure S7C-E), indicating a protective effect against NASH-associated lipid dysregulation.

Additionally, acetate-treated mice exhibited significantly lower serum FITC-dextran and portal vein LPS levels (Figure S8A-B). WB and immunohistochemical analyses further demonstrated that acetate treatment markedly upregulated Claudin-1 and E-cadherin protein expression in colonic tissues (Figure S8C-E).

These findings indicate that acetate suppresses HCC cell proliferation and growth, thereby delaying and mitigating HCC progression.

### APs-CS/PT-NPs suppress the NF-κB signaling pathway to alleviate hepatic fibrosis in HCC

**Fig. 6:**
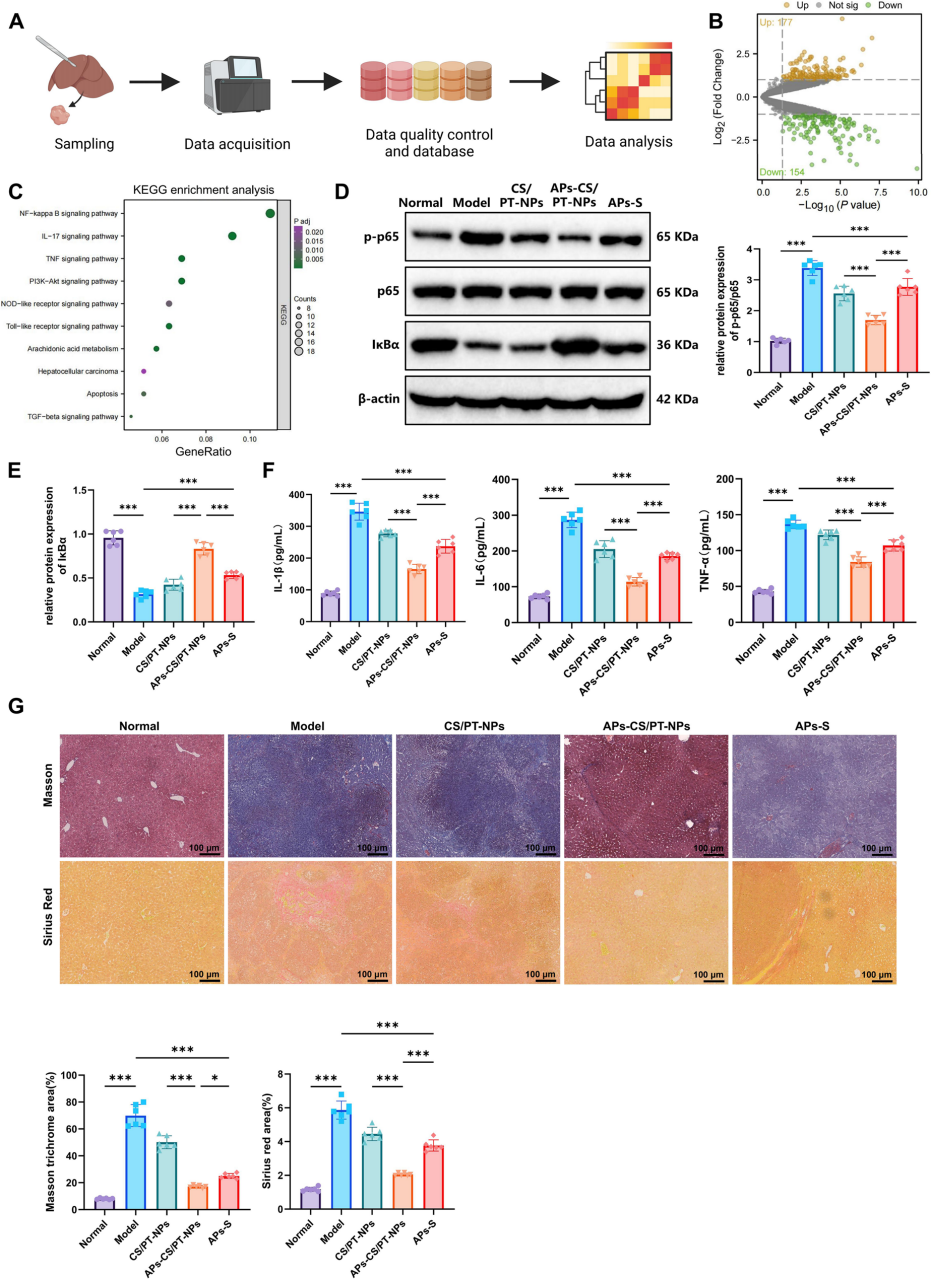
Effects of APs-CS/PT-NPs on Liver Fibrosis Progression in HCC Mice via the NF-κB Signaling Pathway. Note: (**A**) Workflow of transcriptome sequencing; (**B**) Volcano plot of DEGs in liver tissues of APs-CS/PT-NPs and CS/PT-NPs groups (*N* = 3), where yellow dots represent significantly upregulated genes, green dots indicate significantly downregulated genes, and gray dots denote genes with no significant expression differences; (**C**) KEGG enrichment analysis of DEGs; (**D**–**E**) WB analysis of the p-p65/p65 ratio and IκBα protein expression levels in liver tissues; (**F**) ELISA-based quantification of NF-κB downstream target genes IL-6, IL-1β, and TNF-α in liver tissues of HCC mice; (**G**) Masson’s trichrome and Sirius Red staining for fibrosis assessment in liver tissues of HCC mice (scale bar: 100 μm). ***p < 0.001 compared to the control group; *N* = 6

To investigate the molecular mechanisms underlying the inhibitory effects of APs-CS/PT-NPs on HCC progression, we performed RNA sequencing on liver tissues from HCC mice treated with APs-CS/PT-NPs and CS/PT-NPs (Fig. 6A). Differential expression analysis identified 331 DEGs, including 154 downregulated and 177 upregulated genes (Fig. 6B). KEGG pathway enrichment revealed that these DEGs were predominantly associated with the NF-κB, IL-17, TNF, and PI3K–Akt pathways (Fig. 6C). As established in previous studies, NF-κB serves as a central mediator linking hepatic inflammation, fibrosis, and tumorigenesis, representing a critical therapeutic target in liver fibrosis and HCC prevention [64]. Additionally, APs have been reported to modulate GM and alleviate pulmonary fibrosis by inhibiting the TLR4/NF-κB signaling [65].

To validate these transcriptomic findings, WB analysis was conducted to evaluate the expression of key NF-κB signaling components, including p65, phosphorylated p65 (p-p65), and IκBα, in liver tissues. Compared with the Normal group, the Model group exhibited a markedly increased p-p65/p65 ratio and a concomitant decrease in IκBα expression, indicating NF-κB activation. Treatment with APs-S or APs-CS/PT-NPs significantly reduced the p-p65/p65 ratio while restoring IκBα expression, with the most pronounced effect observed in the APs-CS/PT-NPs group (Fig. 6D-E). Similarly, acetate treatment decreased the p-p65/p65 ratio and upregulated IκBα expression in both HCC mouse liver tissues and HCC cell lines (Figure S9A-B). To further confirm the inhibitory effects of APs-CS/PT-NPs and acetate on NF-κB signaling, we measured the expression levels of NF-κB downstream target genes, including IL-6, IL-1β, and TNF-α, in HCC mouse liver tissues via ELISA. The results corroborated the suppression of the NF-κB pathway (Fig. 6F, Figure S9C). Histological assessment using Masson’s trichrome and Sirius Red staining further demonstrated that APs-CS/PT-NPs reduced hepatic fibrosis in HCC mice (Fig. 6G).

In summary, APs-CS/PT-NPs exert antitumor effects in HCC by inhibiting NF-κB signaling activation, thereby mitigating hepatic fibrosis during HCC progression.

### Acetate binds to hepatocyte GPR43 receptor to inhibit the NF-κB signaling pathway and modulate HCC progression

**Fig. 7:**
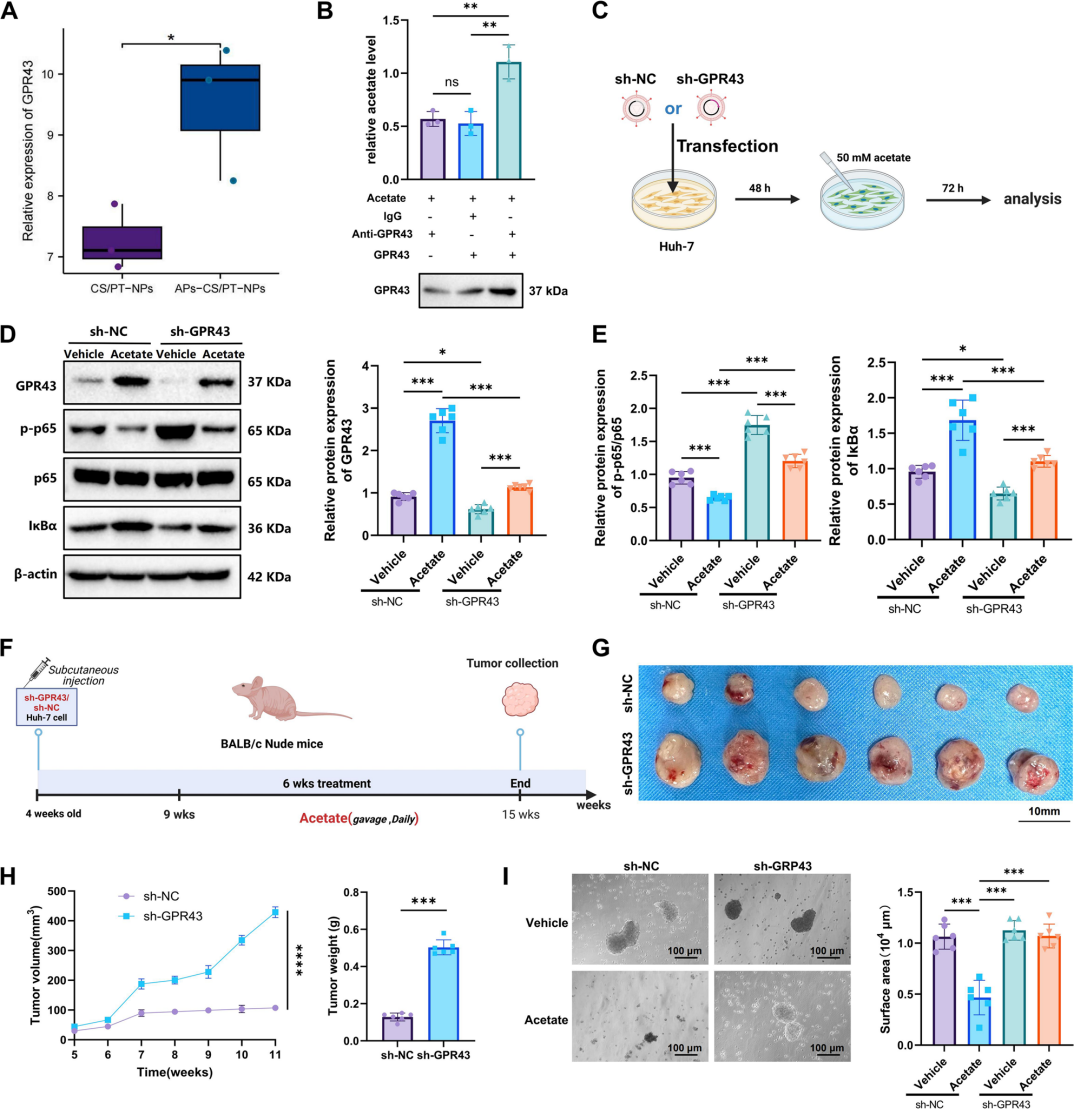
Effects of GPR43/Acetate on HCC Cells and Mice. Note: (**A**) Expression levels of GPR43 protein in transcriptomic data; (**B**) Pulldown assay demonstrating the direct interaction between Acetate and recombinant GPR43 protein; (**C**) Schematic representation of the in vitro experiment: Huh-7 cells were transfected with sh-NC or sh-GPR43-2 lentivirus and treated with Acetate; (**D**–**E**) WB analysis showing changes in GPR43 and NF-κB signaling pathway-related proteins in sh-NC and sh-GPR43 Huh-7 cells after Acetate treatment; (**F**) Schematic representation of the in vivo experiment: Huh-7 cells transfected with sh-GPR43 were subcutaneously xenografted and treated with Acetate; (**G**–**H**) Comparison of tumor volume and weight between the sh-NC and sh-GPR43 groups in xenografted mice; (**I**) GPR43 knockdown in HCC organoids to assess the inhibitory effect of Acetate on organoid growth (scale bar: 100 μm). *Indicates statistical significance between groups: *p < 0.05, **p < 0.01, ***p < 0.001; *N* = 6; in vitro experiments were performed in triplicate

Studies have shown that acetate can bind to the GPR43 protein, and its activation subsequently inhibits NF-κB signaling [66]. WB analysis revealed a significant upregulation of GPR43 protein expression in HCC mouse liver tissues treated with APs-CS/PT-NPs and acetate, as well as in acetate-treated Huh-7 cells (Figure S10A, B, D, E). In contrast, the expression levels of other SCFA receptors, such as GPR41 and GPR109A, in liver tissues remained unchanged following APs-CS/PT-NPs treatment (Figure S10C). Transcriptomic analysis further supported these results, showing a marked increase in hepatic GPR43 expression after APs-CS/PT-NPs treatment (Fig. 7A). To further confirm the interaction between acetate and GPR43, a pull-down assay was performed by co-incubating acetate with recombinant GPR43 protein. The assay results demonstrated that acetate was successfully pulled down by recombinant GPR43, confirming a direct physical binding between acetate and the receptor (Fig. 7B).

To investigate the functional role of GPR43, we transduced Huh-7 cells with lentiviruses carrying sh-NC, sh-GPR43-1, sh-GPR43-2, and sh-GPR43-3 constructs. WB analysis confirmed that sh-GPR43-2 exhibited the highest knockdown efficiency and was thus selected for subsequent experiments (Figure S11A). When acetate was administered to GPR43-deficient Huh-7 cells (Fig. 7C), the inhibitory effect of acetate on NF-κB signaling was significantly attenuated, as evidenced by an increased p-p65/p65 ratio and decreased IκBα expression (Fig. 7D-E). Moreover, GPR43 knockdown reversed the effects of acetate on Huh-7 cell proliferation, apoptosis, and cell cycle arrest (Figure S11B-E).

To further validate that acetate suppresses HCC progression via GPR43, Huh-7 cells transduced with sh-GPR43 were subcutaneously injected into mice for xenograft tumor formation, followed by acetate treatment (Fig. 7F). After six weeks, the sh-GPR43 group displayed significantly larger tumor volumes and weights compared with the sh-NC group (Fig. 7G-H). Additionally, GPR43 knockdown in mouse HCC organoids abolished the inhibitory effect of acetate on organoid growth (Fig. 7I).

Collectively, these findings demonstrate that acetate inhibits the NF-κB signaling pathway by activating the GPR43 receptor, thereby modulating HCC progression.

### GPR43 downregulation reverses the therapeutic effects of APs-CS/PT-NPs on HCC in mice

**Fig. 8:**
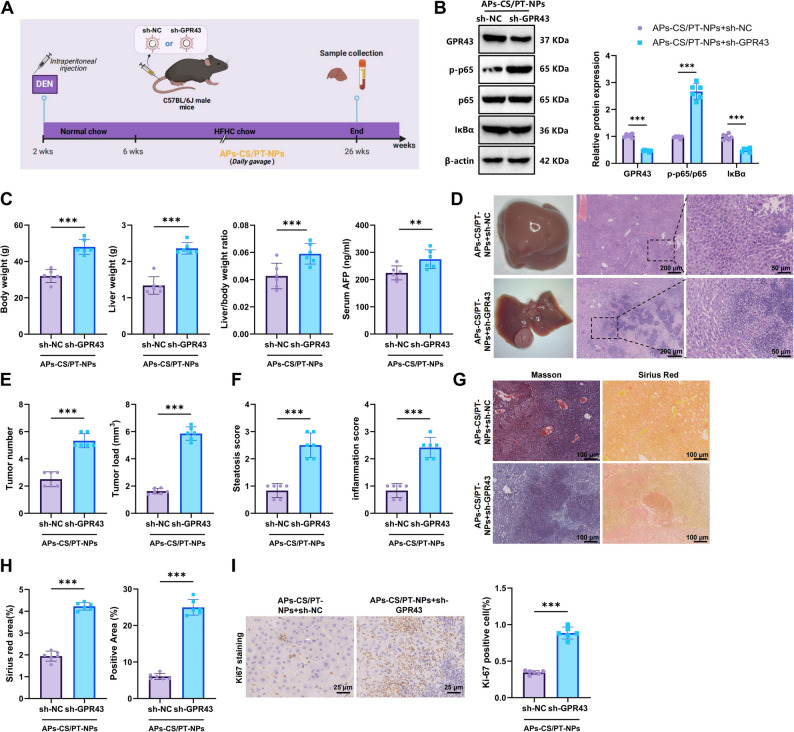
Effects of GPR43 Downregulation on the Therapeutic Efficacy of APs-CS/PT-NPs in HCC Mouse Models. Note: (**A**) Experimental design illustrating dietary intervention and oral administration of APs-CS/PT-NPs in HCC mouse models infected with lentivirus; (**B**) WB analysis of GPR43 expression, p-p65/p65 ratio, and IκBα protein levels in mouse liver tissues; (**C**) Statistical analysis of body weight, liver weight, liver-to-body weight ratio, and serum AFP levels in different groups; (**D**) Representative images of liver morphology and H&E-stained liver tissues (scale bar: 200 μm; enlarged view: scale bar: 50 μm); (**E**) Quantification of tumor number and tumor burden across groups; (**F**) Liver steatosis and inflammation scores in different groups; (**G**-**H**) Masson’s trichrome and Sirius Red staining assessing liver fibrosis in HCC mouse tissues (scale bar: 100 μm); (**I**) Immunohistochemical staining of Ki-67 expression in mouse liver tissues (scale bar: 25 μm). ** indicates *p* < 0.01, *** *p* < 0.001 compared between groups; *N* = 6

To investigate the impact of GPR43 downregulation on the therapeutic efficacy of APs-CS/PT-NPs in HCC, we administered sh-NC and sh-GPR43 lentiviruses via tail vein injection before establishing the NAFLD-HCC mouse model. Concurrently, mice received daily oral gavage of APs-CS/PT-NPs (Fig. 8A). WB analysis revealed that, compared with the APs-CS/PT-NPs + sh-NC group, the APs-CS/PT-NPs + sh-GPR43 group exhibited a markedly increased p-p65/p65 ratio and significantly decreased GPR43 and IκBα expression levels in liver tissues (Fig. 8B). Furthermore, mice in the APs-CS/PT-NPs + sh-GPR43 group displayed significantly higher body weight, liver weight, liver-to-body weight ratio, and serum AFP concentrations compared with the APs-CS/PT-NPs + sh-NC group (Fig. 8C). Histopathological evaluation revealed a substantial increase in tumor burden, hepatic steatosis, inflammatory infiltration, and fibrosis in the GPR43-deficient mice (Fig. 8D-H). Immunohistochemical staining further indicated a significant increase in hepatocyte proliferation in this group (Fig. 8I).

Additionally, serum ALT, AST, and hepatic TG levels were significantly elevated in GPR43-knockdown mice treated with APs-CS/PT-NPs (Figure S12A). Oil Red O staining revealed abundant lipid droplet accumulation in liver tissues of the APs-CS/PT-NPs + sh-GPR43 group (Figure S12B). Moreover, GPR43 downregulation reversed the APs-CS/PT-NPs-induced improvements in glucose tolerance and insulin sensitivity (Figure S12C-D).

These findings indicate that GPR43 downregulation counteracts the therapeutic effects of APs-CS/PT-NPs on HCC in mice.

## Discussion

This study developed an oral colon-targeted delivery system based on APs-CS/PT-NPs and systematically investigated its role in modulating the gut-liver axis, improving GM composition, enhancing acetate levels, and inhibiting the NF-κB signaling pathway. The results demonstrated that APs-CS/PT-NPs effectively reduced hepatic lipid accumulation, alleviated inflammation, mitigated fibrosis, and suppressed the progression of HCC. Mechanistic analyses integrating 16 S rRNA sequencing, targeted metabolomics, and transcriptomics revealed that APs-CS/PT-NPs enhance acetate synthesis, activate the GPR43 receptor, and suppress NF-κB pathway activation, thereby improving the hepatic microenvironment. This study fills an important gap in nanodelivery strategies for HCC prevention and therapy, introducing regulation of the gut–liver axis as a promising early intervention approach. The results provide solid experimental evidence and technological support for precision prevention and treatment of HCC.

The development of HCC is closely linked to dysregulated lipid metabolism, chronic inflammation, and an altered immune microenvironment [67]. Current treatment strategies, including surgical resection, radiotherapy, chemotherapy, molecular-targeted therapies, and immune checkpoint inhibitors, remain limited due to the high heterogeneity and drug resistance of HCC [68, 69]. Increasing evidence has underscored the pivotal role of GM in HCC progression, indicating that modulation of the gut–liver axis represents a promising therapeutic avenue [70, 71]. Microbial dysbiosis has been shown to accelerate tumor development by disturbing bile acid metabolism, activating pro-inflammatory signaling pathways, and reprogramming metabolic networks [72–74]. Building upon this foundation, the present study proposes an innovative strategy that leverages nanotechnology to deliver APs for gut-liver axis modulation. Compared with conventional anti-HCC therapies, APs-CS/PT-NPs exhibit superior targeting capabilities, lower toxicity, and enhanced stability, ensuring precise drug release in the colon and effective microbiota modulation. This novel approach offers a more precise and effective intervention for HCC prevention and treatment.

The GM plays a crucial role in maintaining host health, and its dysbiosis is associated with metabolic abnormalities and immune dysregulation, thereby accelerating the progression of NAFLD to HCC [75–77]. Recent studies have demonstrated significant alterations in the GM of HCC patients, particularly impairments in the SCFA metabolic pathway, leading to reduced anti-inflammatory and metabolic regulatory functions [11, 78–80]. In this study, 16 S rRNA sequencing analysis confirmed that APs-CS/PT-NPs improved the GM composition in HCC mice and enhanced SCFA production. Notably, APs-CS/PT-NPs markedly increased acetate levels, consistent with previous findings and emphasizing the pivotal role of acetate as a key metabolite in HCC prevention and therapy. Unlike conventional probiotic or dietary interventions that indirectly influence GM, the nanoparticle-based targeted delivery of APs achieved enhanced stability and bioavailability in the colon, enabling more precise modulation of microbial balance and expanding the therapeutic potential of microbiota-targeted approaches in HCC.

Previous research has shown that SCFAs regulate inflammation and metabolic homeostasis through GPR signaling pathways [81–83]. The present study further elucidated the molecular mechanism underlying acetate-mediated inhibition of HCC progression, demonstrating that acetate exerts anti-inflammatory and antitumor effects primarily through activation of the GPR43 receptor and suppression of the NF-κB pathway. WB and transcriptomic analyses demonstrated that APs-CS/PT-NPs treatment significantly upregulated GPR43 expression while reducing the activity of key NF-κB pathway proteins, including phosphorylated IκBα (p-IκBα) and NF-κB p65. These findings indicate that acetate suppresses NF-κB-mediated pro-inflammatory responses via the GPR43 signaling axis. While previous studies have reported similar mechanisms, this study is the first to propose a nanoparticle-based delivery strategy to enhance acetate levels, thereby achieving targeted modulation of the GPR43 signaling axis for HCC prevention and treatment. This novel approach provides new insights into immunometabolic regulation in HCC therapy.

The development of HCC is strongly influenced by the tumor microenvironment (TME), characterized by elevated levels of pro-inflammatory cytokines (e.g., IL-6, TNF-α) and an enhanced immunosuppressive microenvironment contribute to HCC progression [84–86]. The findings demonstrate that APs-CS/PT-NPs markedly reduce hepatic pro-inflammatory cytokine levels, mitigate lipid accumulation, and alleviate hepatic fibrosis, thereby improving the overall HCC microenvironment. In contrast to conventional anti-inflammatory or anti-tumor therapies, the approach regulates the gut–liver axis to indirectly reshape hepatic metabolism and inflammatory status, providing a systemic therapeutic framework consistent with the multifactorial nature of HCC. This strategy broadens the application of microenvironmental modulation in HCC therapy.

Nanomedicine-based drug delivery systems have been widely utilized in cancer treatment [87, 88]; however, nanoparticle-based strategies targeting the gut-liver axis and GM remain limited. In this study, a colon-targeted APs-CS/PT-NP delivery system was designed to enhance drug stability, bioavailability, and precision targeting. Through this nanoparticle-based approach, the therapeutic efficacy of APs in HCC treatment was significantly improved. Our experiments further revealed that the negatively charged APs strongly interact electrostatically with the CS/PT matrix, suppressing premature APs release and enabling the NPs to pass through the stomach and upper gastrointestinal tract intact. In the lower intestine, CS is deprotonated, reducing the electrostatic attraction between CS and PT compared to the upper GI tract. In addition, the GM facilitates the degradation of both CS and PT. This combination of microbe-mediated degradation and weakened electrostatic forces leads to nanoparticle disintegration and the burst release of APs in the colon. These findings suggest that CS/PT NPs are promising carriers for colon-targeted APs delivery, as their coating integrity, electrostatic stability, and limited swelling under acidic conditions enable them to transit through the upper GI tract. The unique features of APs-CS/PT-NPs may also protect the active compound from enzymatic degradation in the intestine, minimize unwanted interactions with the small intestinal mucosa, and potentially enhance therapeutic efficacy while reducing irritation to the upper gastrointestinal tract. Compared with conventional drug delivery methods, the nanocarrier system achieves superior drug targeting and lower systemic toxicity, providing a new perspective on nanotechnology-driven strategies for HCC therapy.

Despite these promising results, this study has certain limitations. First, as the research primarily relies on murine models, the lack of large-scale clinical validation necessitates further clinical trials to confirm the efficacy of APs-CS/PT-NPs in HCC treatment. Second, the long-term safety profile and potential toxicological effects of APs-CS/PT-NPs require additional preclinical and clinical studies to evaluate their sustained impact. Third, although the study focused on the NF-κB signaling pathway, the pathogenesis of HCC involves multiple molecular mechanisms. Future research should explore the broader regulatory effects of APs-CS/PT-NPs on additional signaling pathways to enhance their therapeutic potential. In terms of material synthesis and formulation, the current work emphasized proof-of-concept development and performed only preliminary optimization of formulation parameters, primarily balancing electrostatic interactions and encapsulation efficiency. Further studies could adopt a design of experiments (DoE) framework to refine the formulation more systematically. Evaluation of scalability and batch-to-batch reproducibility will be essential for clinical translation; however, these experiments were not conducted due to laboratory constraints and will be prioritized in subsequent research. The GM analysis also presents certain constraints. The results were derived from a single mouse cohort and specific time points. Although adequate biological replicates were included and statistical analyses consistently demonstrated significant trends in α/β diversity, Bifidobacterium abundance, and acetate or acetate ester levels, independent validation across multiple cohorts and time points remains necessary. Consequently, the current conclusions primarily reflect stable observations under defined experimental conditions. Future studies will incorporate independent animal cohorts and longitudinal sampling to strengthen reproducibility, generalizability, and translational relevance.

## Conclusion

Based on the findings of this study, we draw the following preliminary conclusions: APs, formulated as APs-CS/PT NPs, enable targeted delivery to the colon, effectively modulate GM composition and improve gut-liver axis function. Through these mechanisms, the formulation reduces hepatic lipid accumulation, inhibits fibrosis, and prevents the malignant transformation associated with HCC. In addition, APs-CS/PT-NPs enhance intestinal barrier integrity and exert antitumor effects by regulating SCFA levels (e.g., acetate) and suppressing the NF-κB signaling pathway. The findings provide a theoretical foundation for the development of gut-targeted nanodelivery systems and offer an alternative therapeutic approach for HCC. Moreover, the results highlight the potential of APs in modulating liver pathology through GM regulation and gut–liver axis restoration, underscoring the pivotal role of the intestinal microenvironment in HCC prevention and therapy. Leveraging the gut–liver axis as a therapeutic target may enable a more precise and holistic treatment strategy, offering new insights for managing HCC and other diseases linked to gut dysbiosis.

## Supplementary Information

Supplementary Material 1

Supplementary Material 2

Supplementary Material 3

Supplementary Material 4

Supplementary Material 5

Supplementary Material 6

Supplementary Material 7

Supplementary Material 8

Supplementary Material 9

Supplementary Material 10

Supplementary Material 11

Supplementary Material 12

Supplementary Material 13

## Data Availability

All data can be provided as needed.

## Abbreviations

AFP: Alpha-Fetoprotein
APs: Astragalus Polysaccharide
APs-CS/PT-NPs: Astragalus Polysaccharide-Loaded Chitosan/Pectin Nanoparticles
AR: Astragalus Membranaceus
AST: Aspartate Aminotransferase
ALT: Alanine Aminotransferase
BUN: Blood Urea Nitrogen
BP: Biological Process
BSA: Bovine Serum Albumin
CRE: Creatinine
CPCoA: Constrained Principal Coordinates Analysis
CC: Cellular Component
DMSO: Dimethyl Sulfoxide
DEGs: Differentially Expressed Genes
DLS: Dynamic Light Scattering
EE: Encapsulation Efficiency
FITC: Fluorescein Isothiocyanate
FBS: Fetal Bovine Serum
GO: Gene Ontology
GPR43: G-Protein-Coupled Receptor 43
GPRs: G-Protein-Coupled Receptors
GTT: Glucose Tolerance Test
H&E: Hematoxylin and Eosin
HFD: High-Fat Diet
HCC: Hepatocellular Carcinoma
HRP: Horseradish Peroxidase
IHC: Immunohistochemistry
IPGTT: Intraperitoneal Glucose Tolerance Test
ITT: Insulin Tolerance Test
KEGG: Kyoto Encyclopedia of Genes and Genomes
LDA: Linear Discriminant Analysis
LC-MS: Liquid Chromatography-Mass Spectrometry
LEfSe: Linear Discriminant Analysis Effect Size
LPS: Lipopolysaccharides
MTT: 3-(4,5-Dimethylthiazol-2-yl)-2,5-Diphenyltetrazolium Bromide
MF: Molecular Function
NF-κB: Nuclear Factor Kappa B
NAFL: Non-Alcoholic Fatty Liver
NAFLD: Non-Alcoholic Fatty Liver Disease
NAFLD-HCC: Non-Alcoholic Fatty Liver Disease-Hepatocellular Carcinoma
OCDDS: Oral Colon Targeted Delivery Systems
PFA: Paraformaldehyde
PBS: Phosphate-Buffered Saline
PI: Propidium Iodide
SCFAs: Short-Chain Fatty Acids
TG: Triglyceride
TEM: Transmission Electron Microscope
TME: Tumor Microenvironment
TPP: Tripolyphosphate
WB: Western Blot
