# Supplementary figures and images for: Colon-Targeted astragalus polysaccharide nanoparticles prevent NAFLD-Driven hepatocarcinogenesis via microbiota remodeling and NF-κB Inhibition
Source: J Exp Clin Cancer Res. 2025 Dec 20;44:330. doi: 10.1186/s13046-025-03608-z (PMC12751668; doi:10.1186/s13046-025-03608-z)

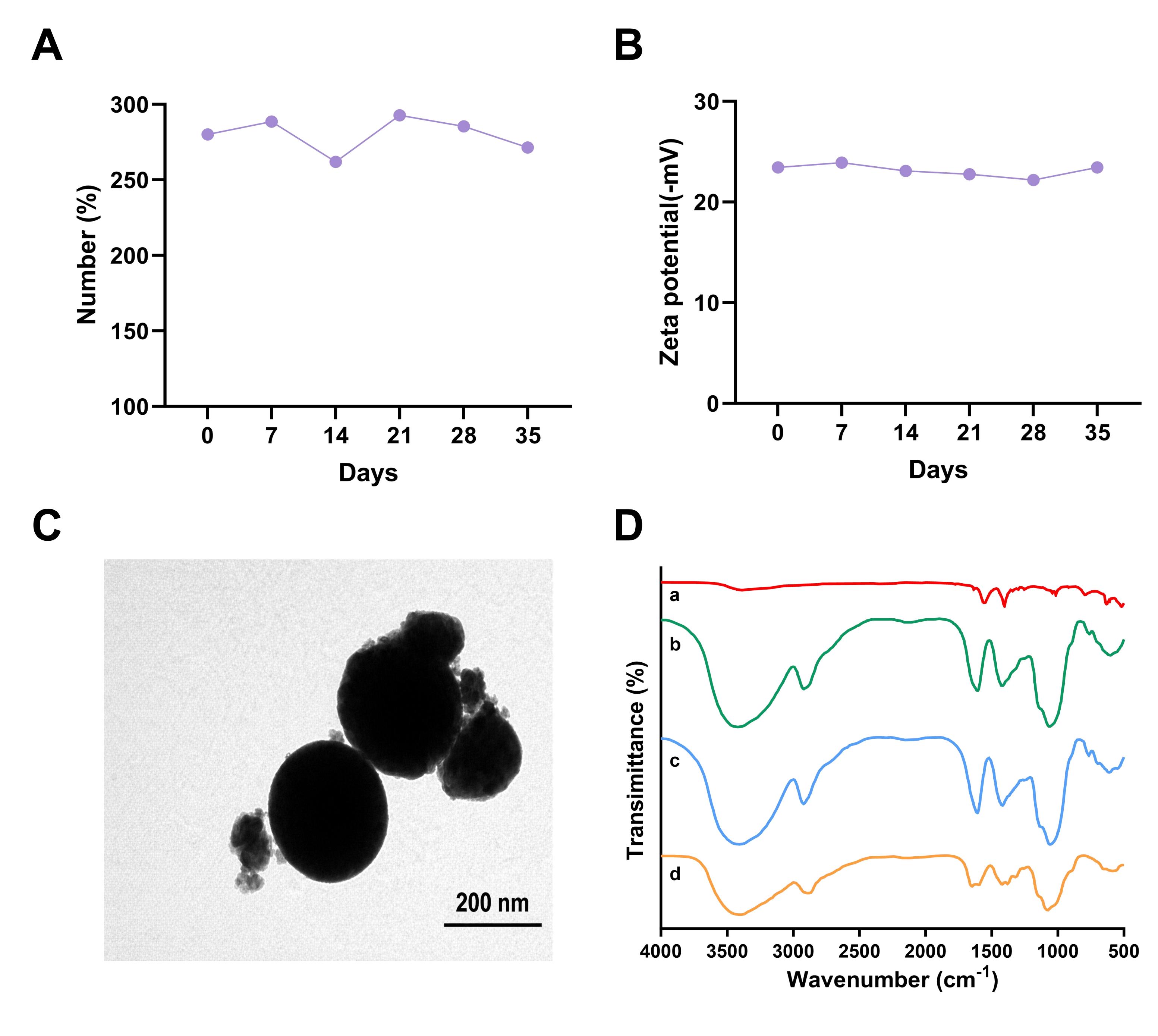

Supplement: Supplementary file 1 — Supplementary Material 1 [file 13046_2025_3608_MOESM1_ESM.jpg]

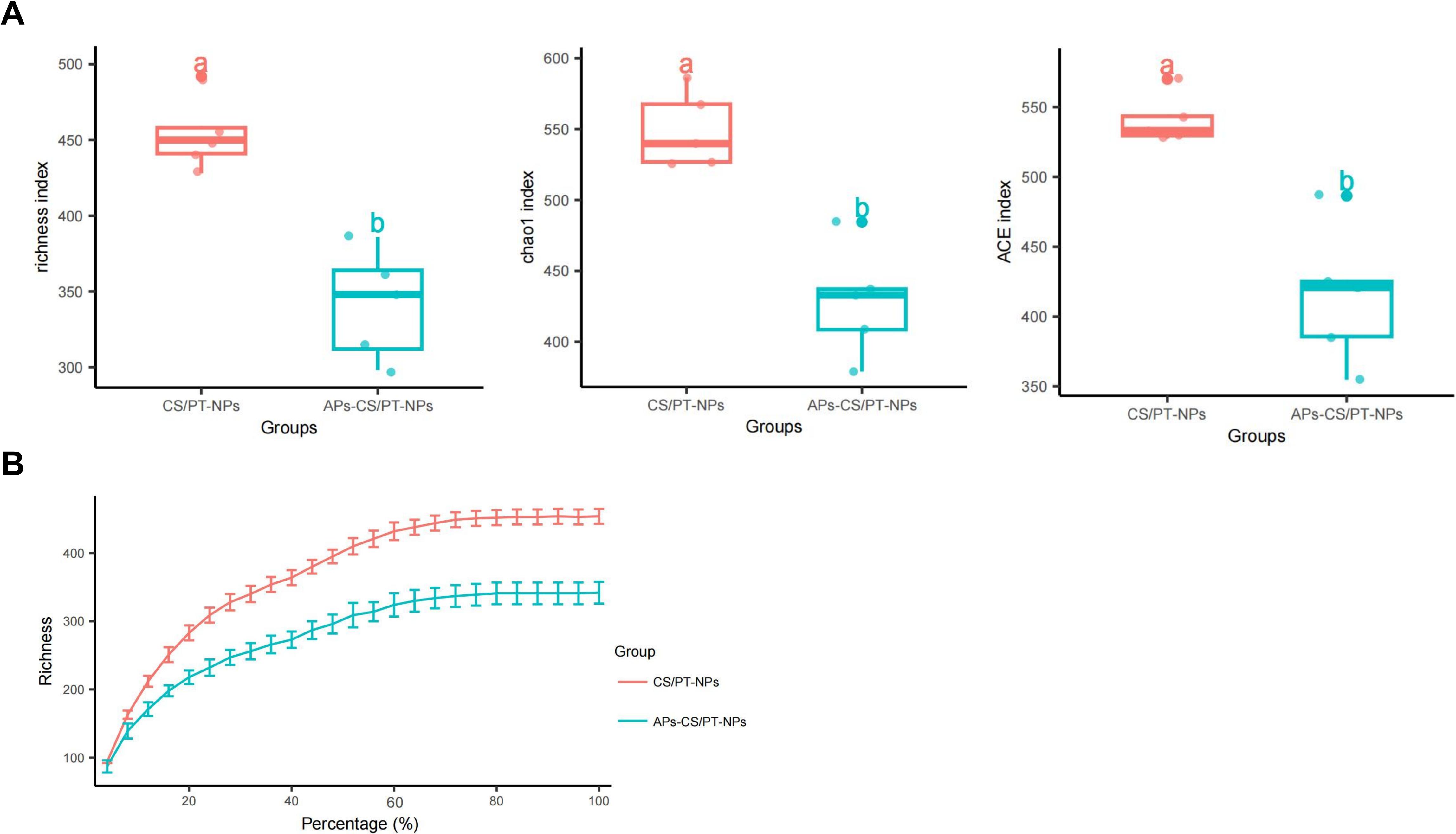

Supplement: Supplementary file 2 — Supplementary Material 2 [file 13046_2025_3608_MOESM2_ESM.jpg]

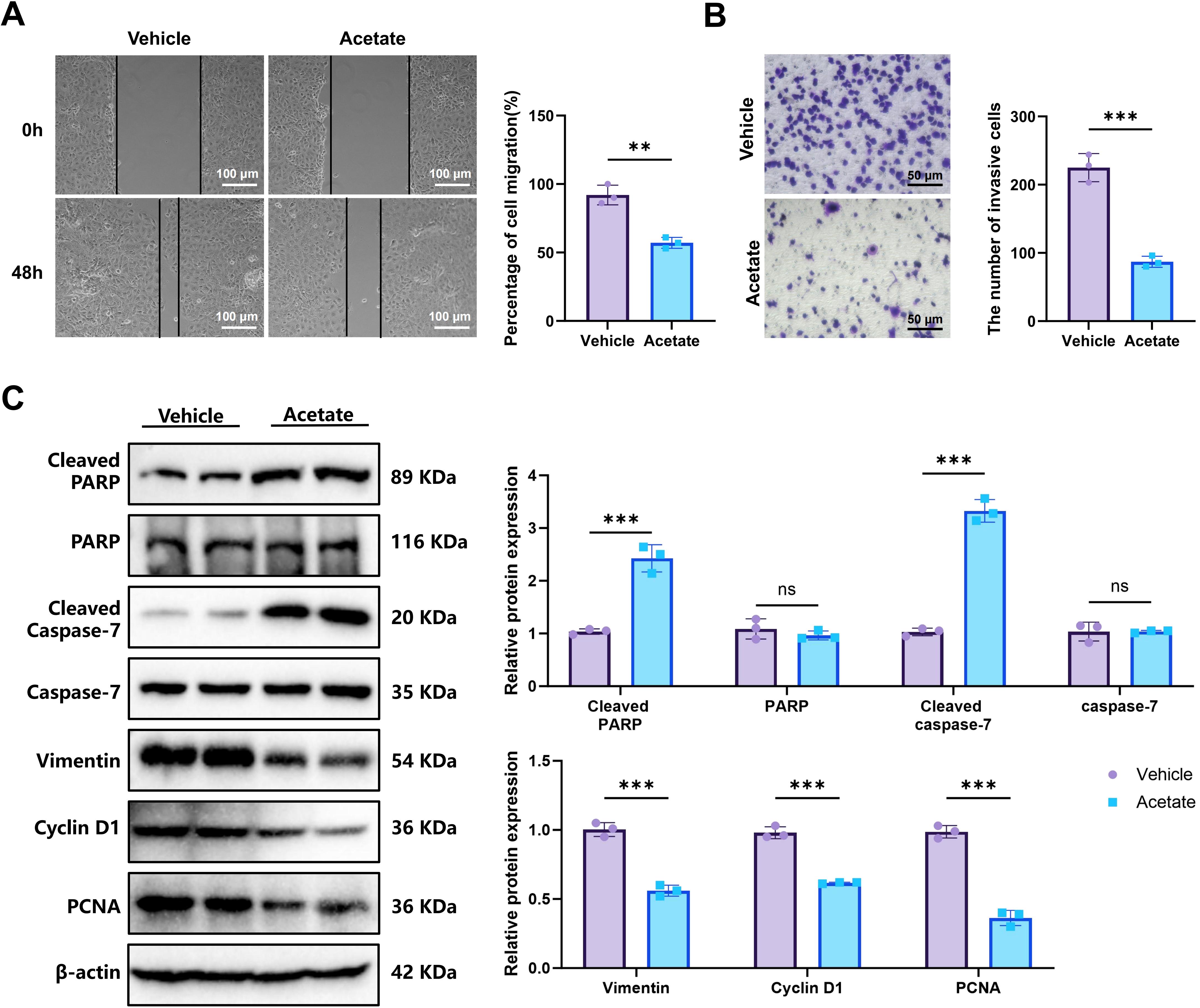

Supplement: Supplementary file 3 — Supplementary Material 3 [file 13046_2025_3608_MOESM3_ESM.jpg]

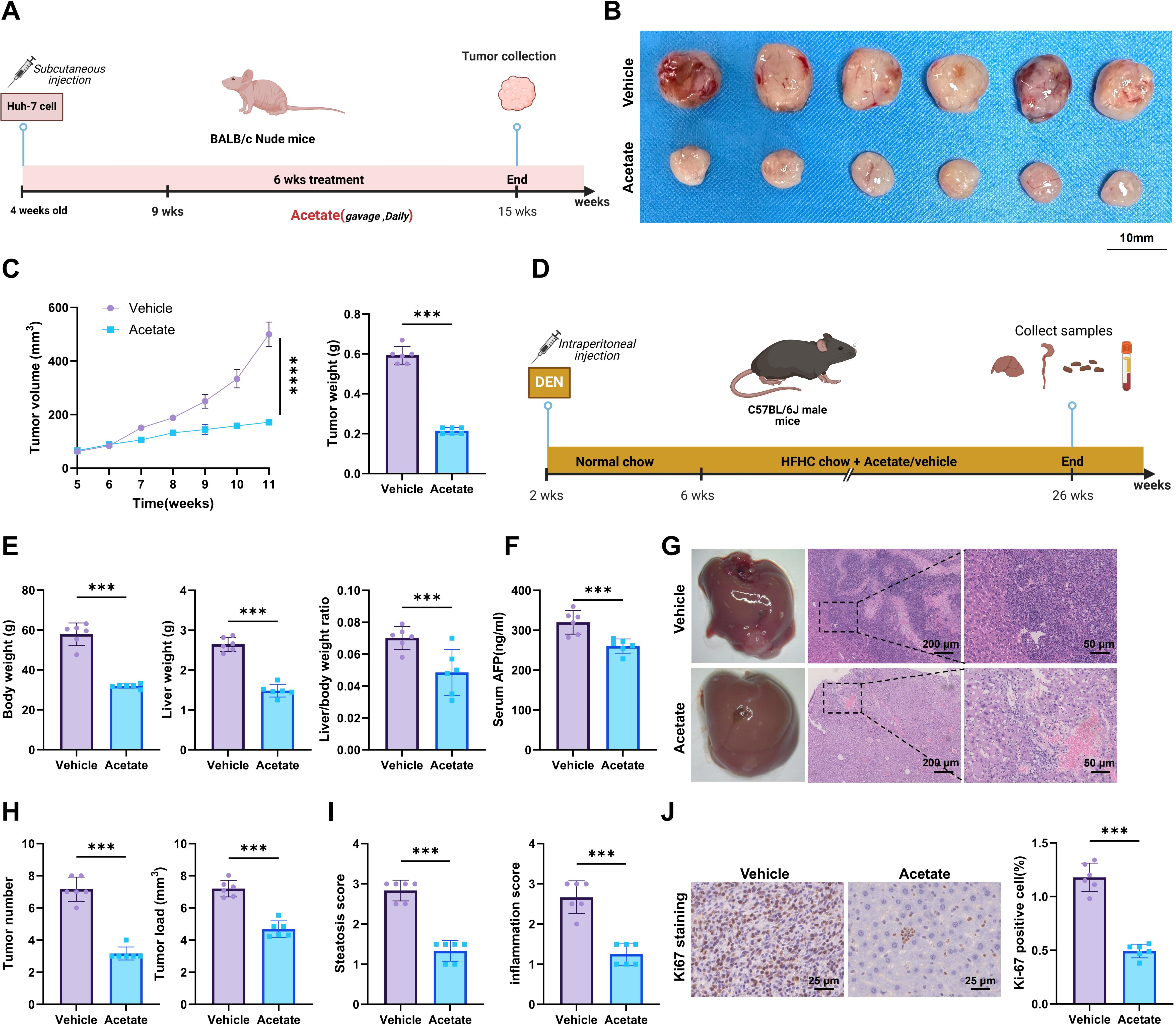

Supplement: Supplementary file 4 — Supplementary Material 4 [file 13046_2025_3608_MOESM4_ESM.jpg]

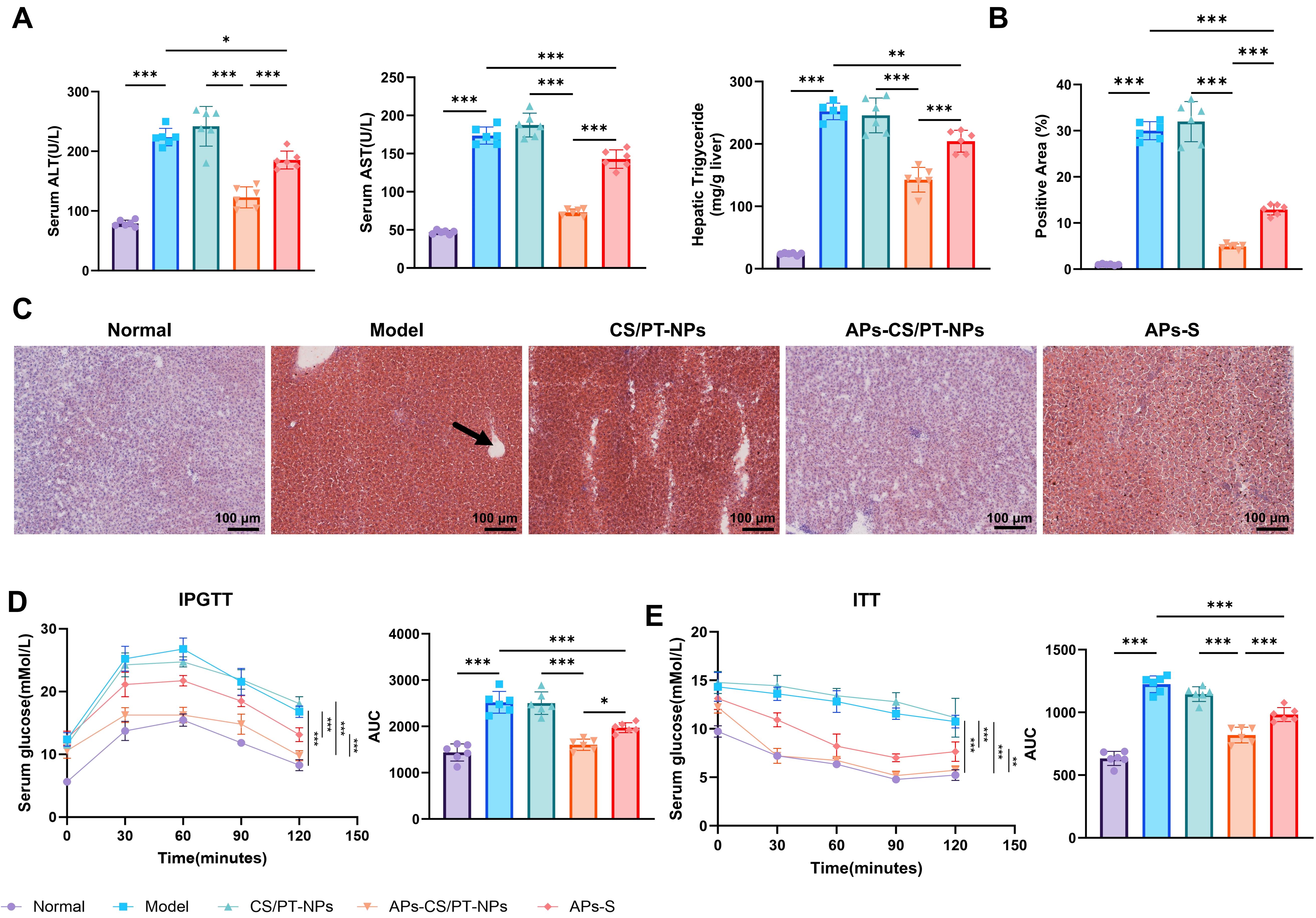

Supplement: Supplementary file 5 — Supplementary Material 5 [file 13046_2025_3608_MOESM5_ESM.jpg]

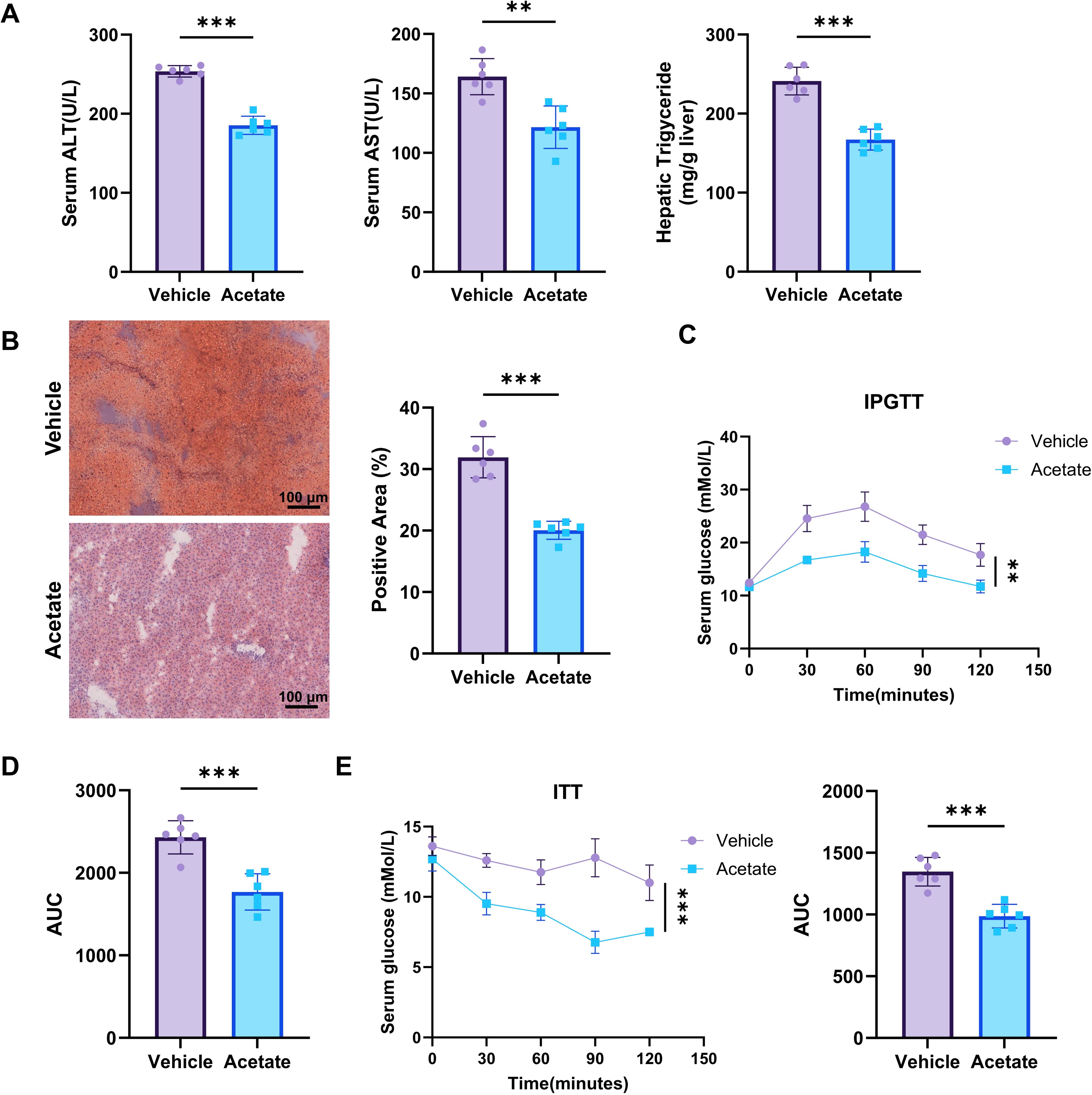

Supplement: Supplementary file 6 — Supplementary Material 6 [file 13046_2025_3608_MOESM6_ESM.jpg]

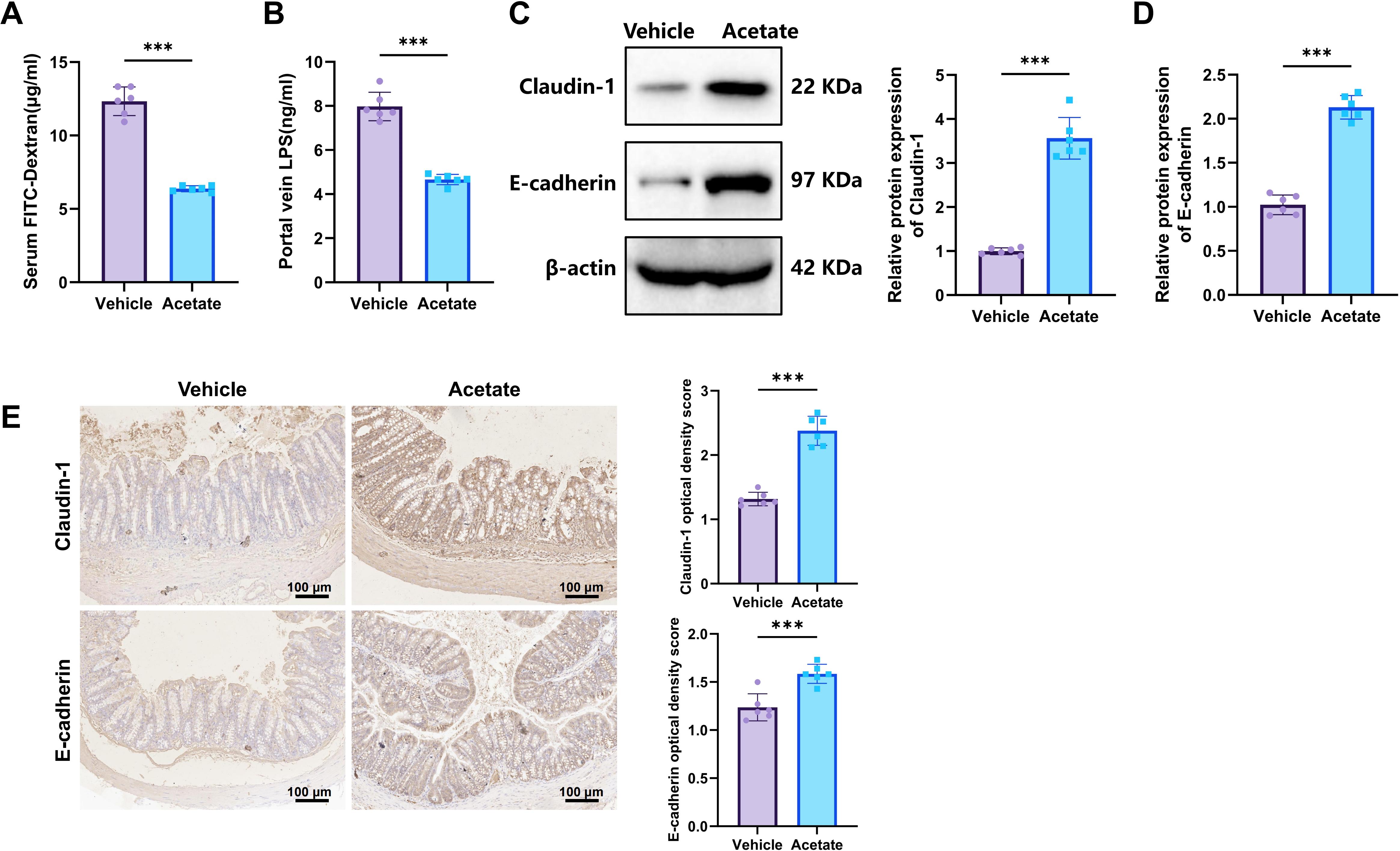

Supplement: Supplementary file 7 — Supplementary Material 7 [file 13046_2025_3608_MOESM7_ESM.jpg]

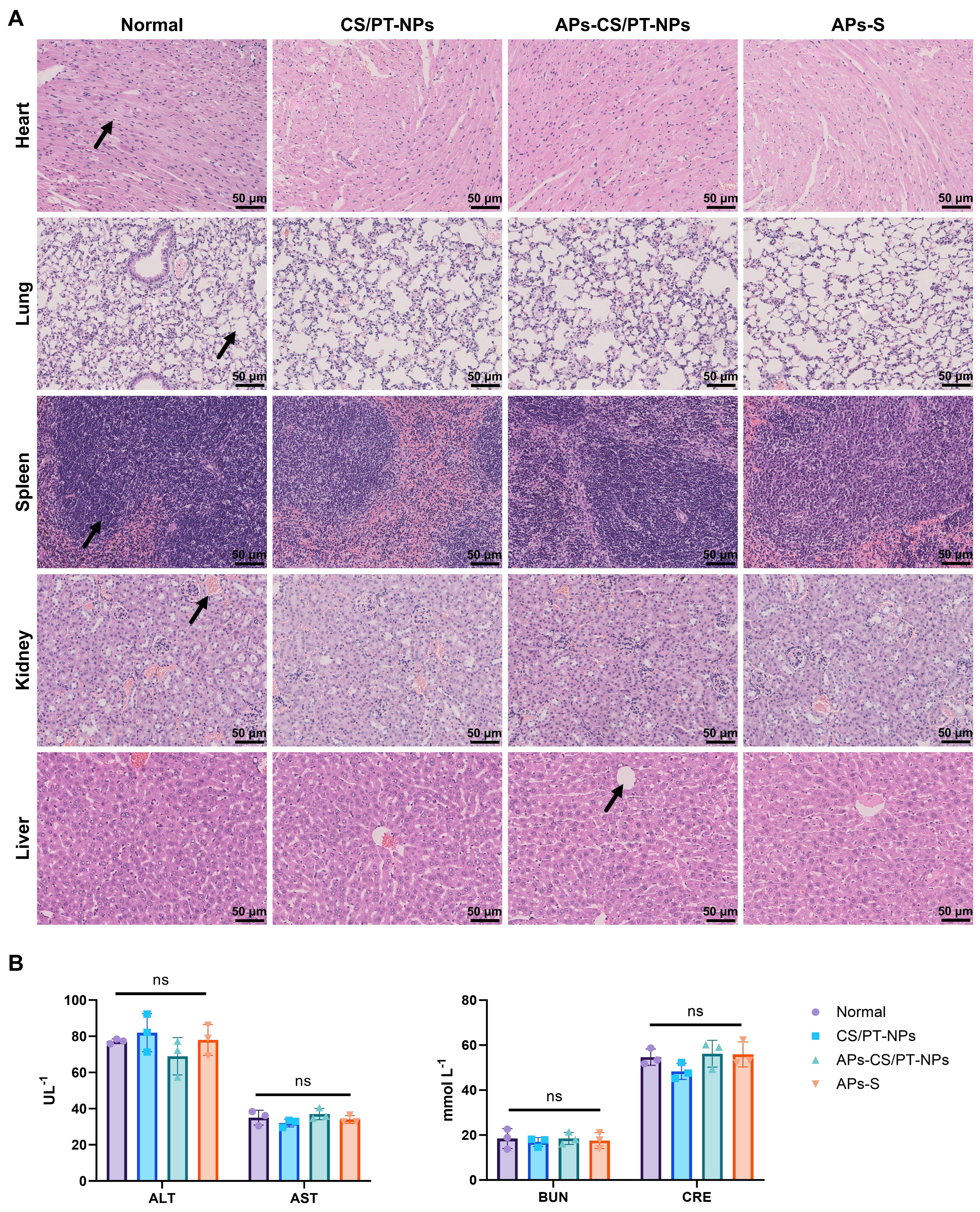

Supplement: Supplementary file 8 — Supplementary Material 8 [file 13046_2025_3608_MOESM8_ESM.jpg]

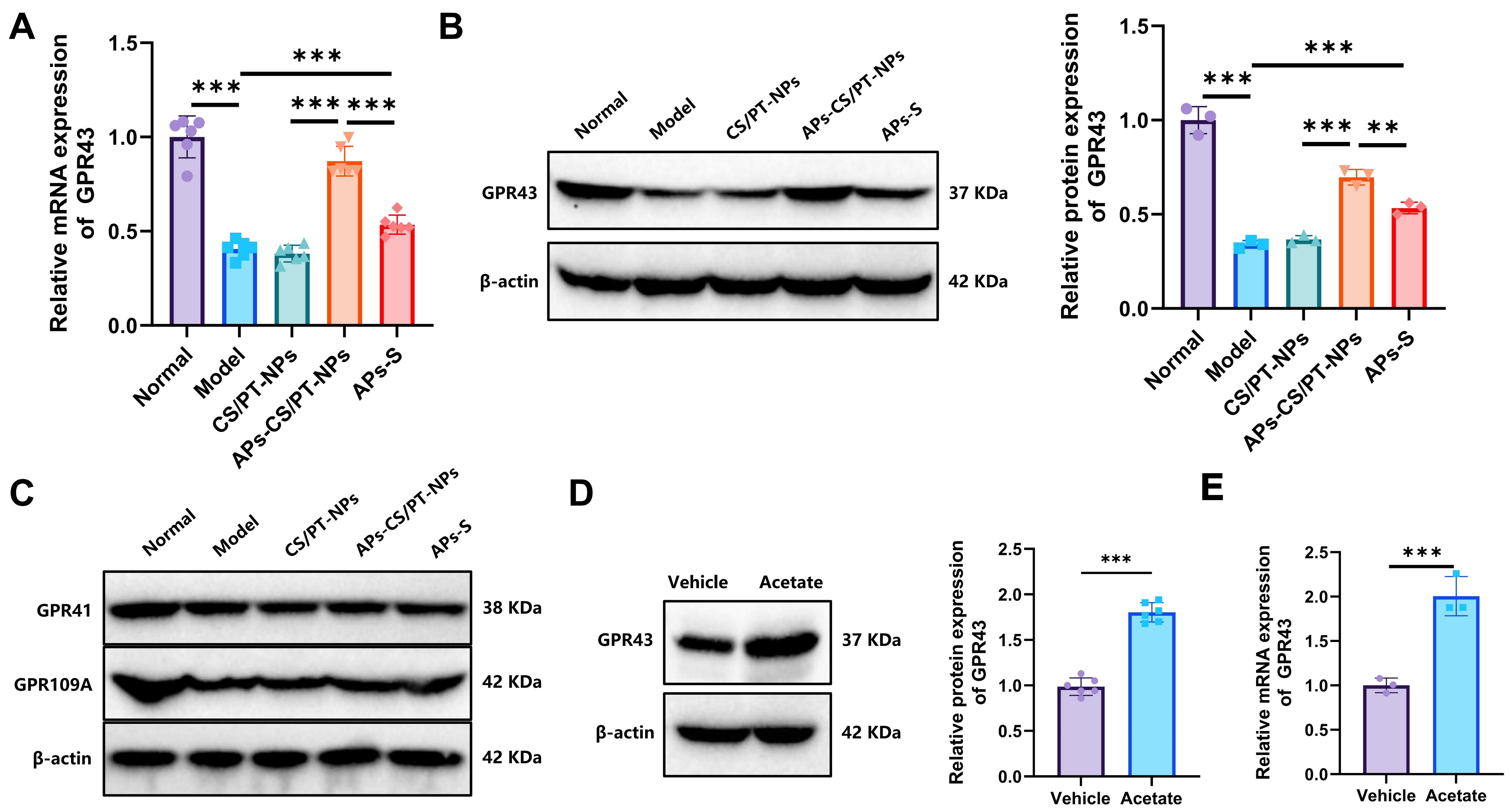

Supplement: Supplementary file 9 — Supplementary Material 9 [file 13046_2025_3608_MOESM9_ESM.jpg]

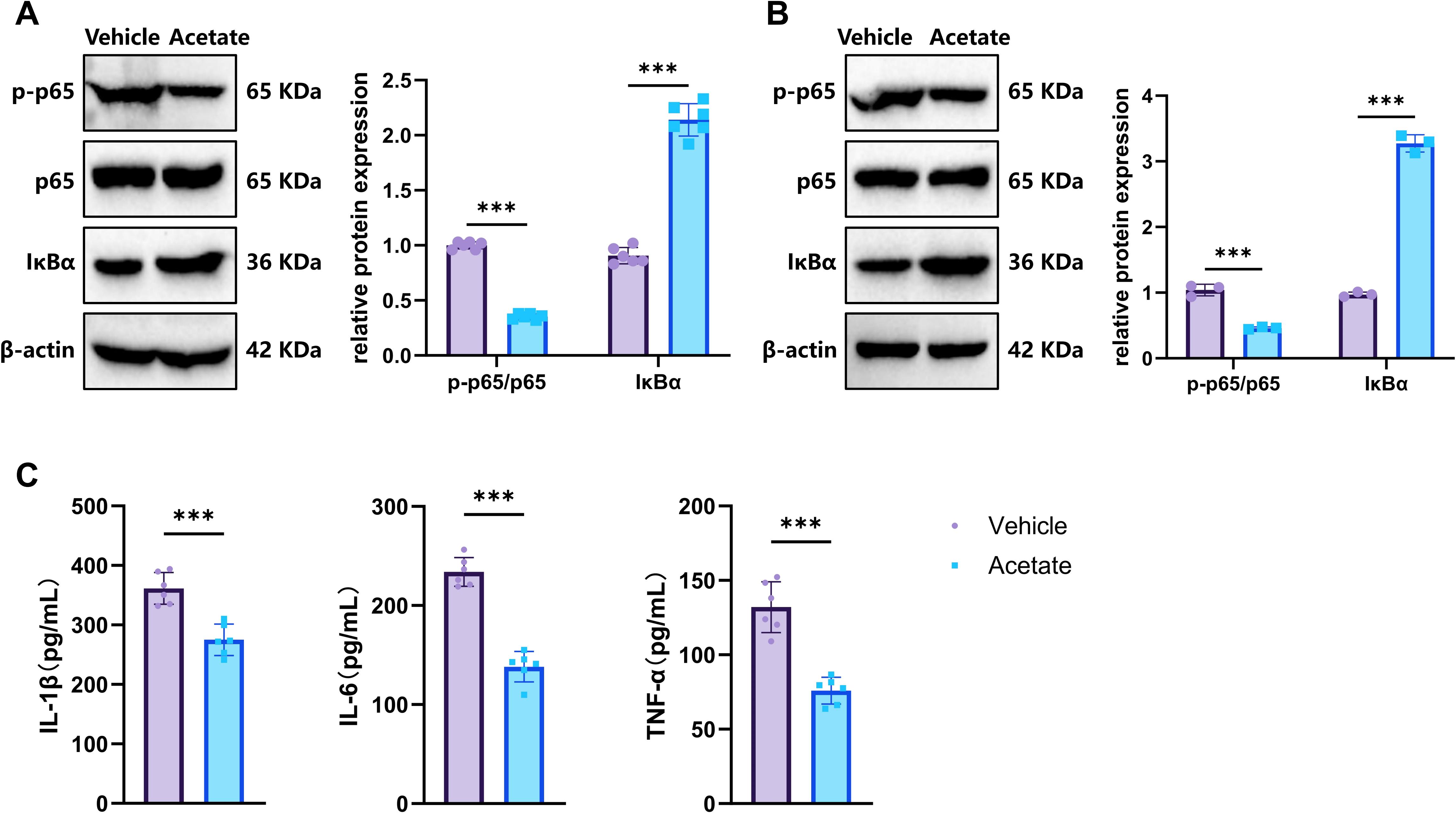

Supplement: Supplementary file 10 — Supplementary Material 10 [file 13046_2025_3608_MOESM10_ESM.jpg]

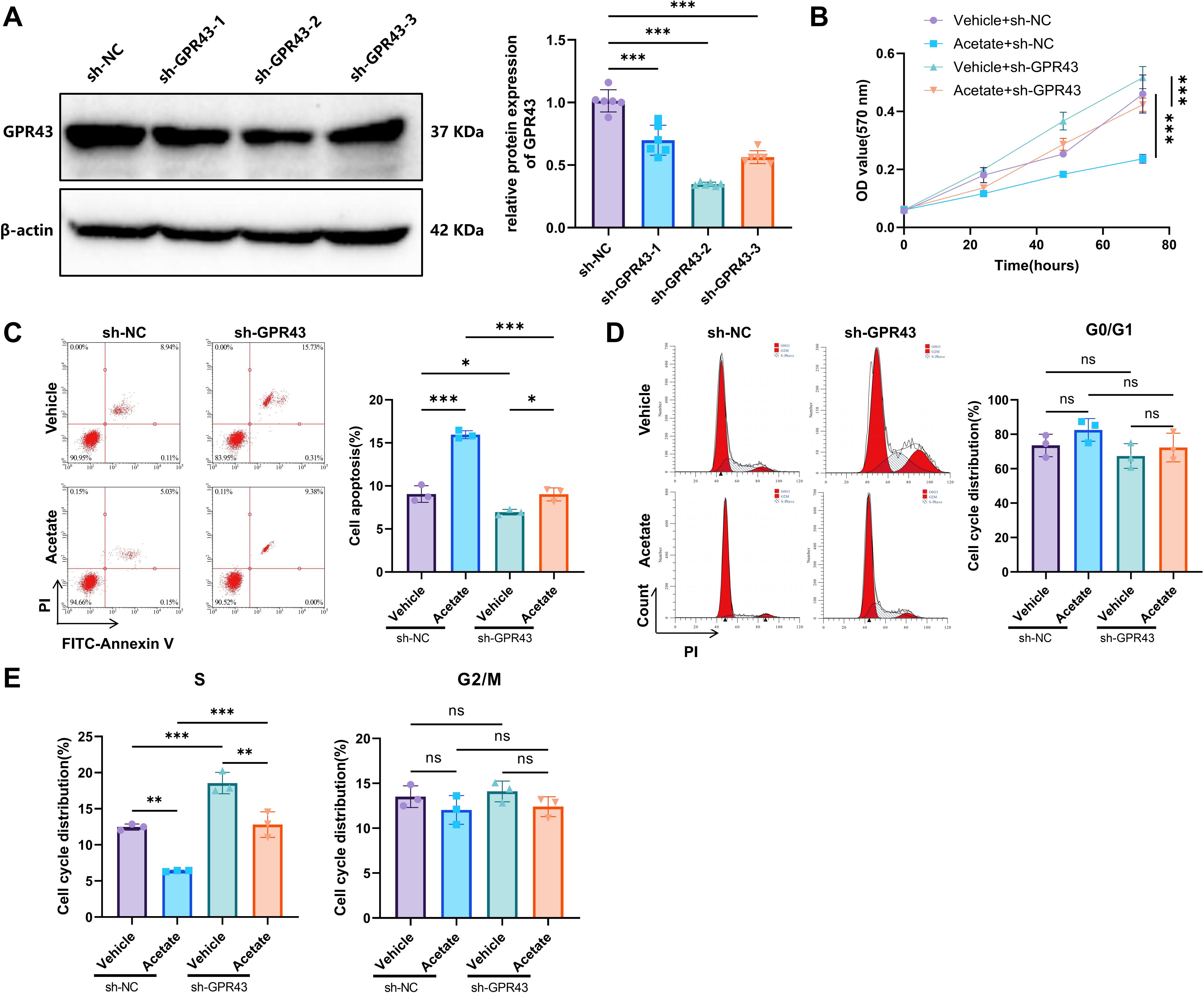

Supplement: Supplementary file 11 — Supplementary Material 11 [file 13046_2025_3608_MOESM11_ESM.jpg]

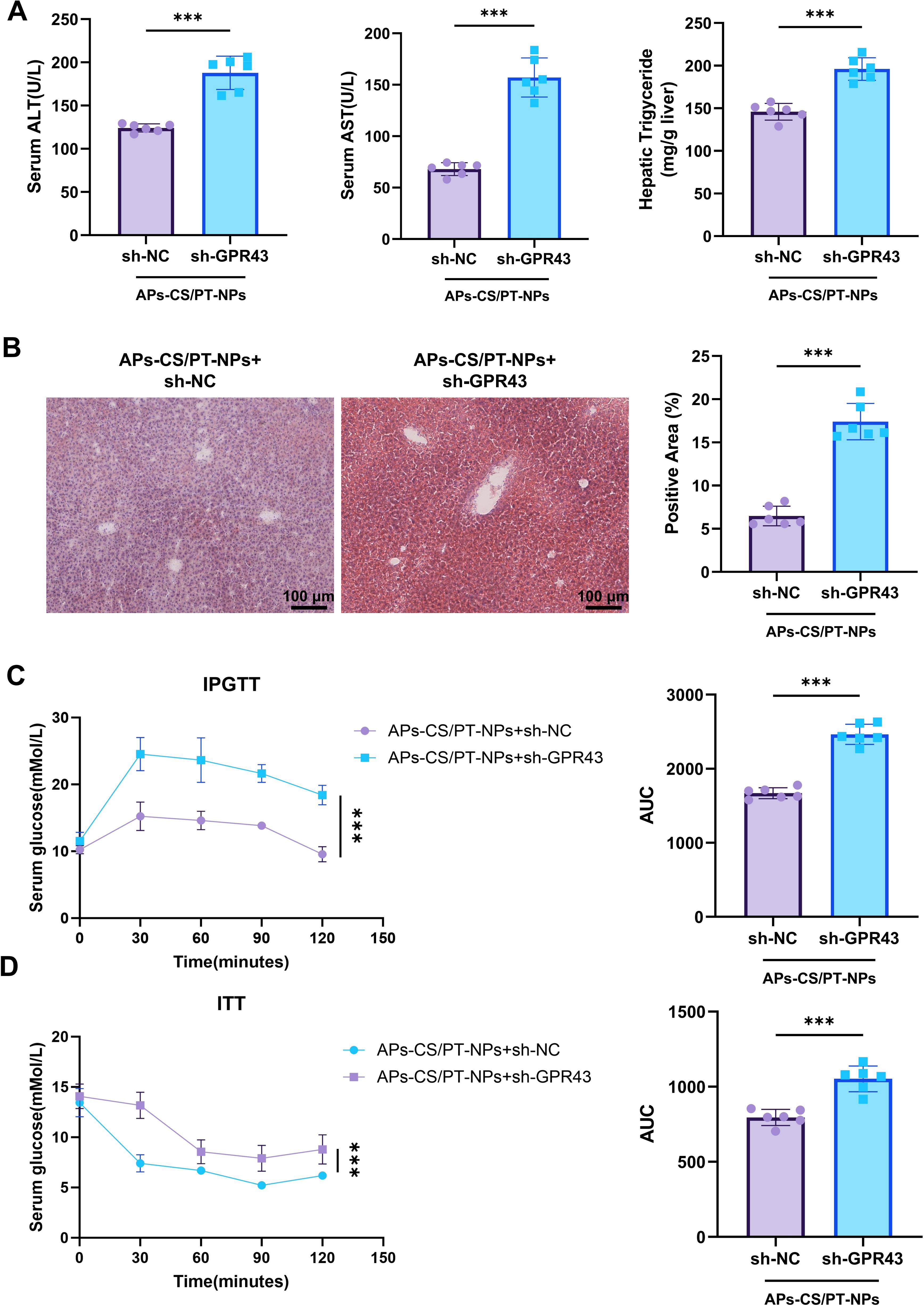

Supplement: Supplementary file 12 — Supplementary Material 12 [file 13046_2025_3608_MOESM12_ESM.jpg]
