# Supplementary material for: Colon-Targeted astragalus polysaccharide nanoparticles prevent NAFLD-Driven hepatocarcinogenesis via microbiota remodeling and NF-κB Inhibition
Source: J Exp Clin Cancer Res. 2025 Dec 20;44:330. doi: 10.1186/s13046-025-03608-z (PMC12751668; doi:10.1186/s13046-025-03608-z)
Supplement: Supplementary file 13 — Supplementary Material 13 [file 13046_2025_3608_MOESM13_ESM.docx]

**Table S1. GPR43 Interference Sequence.**

| **Targets** | **Sequences** |
| --- | --- |
| sh-NC | 5'-CCTAAGGTTAAGTCGCCCTCG-3' |
| sh-GPR43-1(human) | 5'-CGTGGTGTTCAGTTCACTCAA-3' |
| sh-GPR43-2(human) | 5'-GATCCTCATGGCTTACATCAT-3' |
| sh-GPR43-3(human) | 5'-ACTGAATTGTCCTACTCAAAG-3' |

**Table S2. Primary Antibody Manufacturer Information.**

| **Antibody name** | **Antibody item number** | **concentration** | **manufacturer** |
| --- | --- | --- | --- |
| Anti-Claudin 1 | ab307692 | 1：1000 | Abcam |
| Anti-E-Cadherin | ab314063 | 1：1000 | Abcam |
| Anti-PARP | 9532 | 1：1000 | Cell Signaling Technology |
| Anti-Cleaved PARP | 5625 | 1：1000 | Cell Signaling Technology |
| Anti-Caspase-7 | 9492 | 1：1000 | Cell Signaling Technology |
| Anti-Cleaved Caspase-7 | 9491 | 1：500 | Cell Signaling Technology |
| Anti-Vimentin | 5741 | 1：1000 | Cell Signaling Technology |
| Anti-Cyclin D1 | 2922 | 1：1000 | Cell Signaling Technology |
| Anti-PCNA | 13110 | 1：1000 | Cell Signaling Technology |
| Anti-NF-κB p65 | 8242 | 1：1000 | Cell Signaling Technology |
| Anti-Phospho-NF-κB p65 | 3033 | 1：1000 | Cell Signaling Technology |
| Anti-IκBα | 10268-1-AP | 1：1000 | Proteintech |
| Anti-GPR43 | 84544-1-RR | 1：1000 | Proteintech |
